# Supplementary material for: HIV-1-induced nuclear invaginations mediated by VAP-A, ORP3, and Rab7 complex explain infection of activated T cells
Source: Nat Commun. 2023 Aug 10;14:4588. doi: 10.1038/s41467-023-40227-8 (PMC10415338; doi:10.1038/s41467-023-40227-8)
Supplement: Supplementary file 1 — Supplementary Information [file 41467_2023_40227_MOESM1_ESM.pdf]

**Supplementary Information**

**Title:**

HIV-1-induced nuclear invaginations mediated by VAP-A, ORP3, and Rab7 complex explain infection of activated T cells

**Author list:**

Mark F. Santos[1], Germana Rappa[1], Jana Karbanová[2], Patrizia Diana[3], Girolamo Cirrincione[3], Daniela Carbone[3], David Manna[4], Feryal Aalam[1], David Wang[1], Cheryl Vanier[1, §], Denis Corbeil[2] and Aurelio Lorico[1]

**Affiliation:**

[1] Touro University Nevada College of Osteopathic Medicine; Henderson, Nevada, USA

[2] Biotechnology Center (BIOTEC) and Center for Molecular and Cellular Bioengineering, Technische Universität Dresden; Dresden, Germany

[3] Department of Biological, Chemical, and Pharmaceutical Sciences and Technologies (STEBICEF), University of Palermo, Palermo, Italy

[4] Touro College of Osteopathic Medicine, Middletown; New York, USA

[§] Present address: Imgen Research, LLC, 5495 South Rainbow #201, Las Vegas, NV 89118, USA

**Corresponding authors:**

Denis Corbeil: denis.corbeil@tu-dresden.de

Aurelio Lorico: alorico@touro.edu

**Content:**

| | |
|---|---|
| Supplementary Fig. 1. | Gag-iGFP and HIV-89.6-EGFP signals in viral particles. |
| Supplementary Fig. 2. | Colocalization of HIV-1 IN and Gag-iGFP in NEI. |
| Supplementary Fig. 3. | HIV-Gag-iGFP infection increases the number of type II NEI. |
| Supplementary Fig. 4. | Viral HIV-1 p24 antigen is associated with Rab7$^+$ late endosomes. |
| Supplementary Fig. 5. | Silencing VAP-A, VAP-B and ORP3 in HeLa cells. |
| Supplementary Fig. 6. | HIV-1 integrase enters the NR *via* late endosomes with a restricted localization at the perinuclear region. |
| Supplementary Fig. 7. | Impact of PRR851 concentration on Rab7 binding to ORP3, entry of HIV-1 IN in NEIs and productive infection. |
| Supplementary Fig. 8. | Evaluation of drug toxicity on HeLa and primary CD4$^+$ T cells. |

| | |
|---|---|
| Supplementary Note 1. | Information relative to HeLa cells infected with HIV-1 virus containing native Env. |
| Supplementary Fig. 9. | The VOR complex inhibition by itraconazole or PRR851 impedes the HIV-1-associated EGFP expression. |
| Supplementary Fig. 10. | Cell surface CD4 stimulates the uptake of HIV-1 components in HeLa cells. |
| Supplementary Fig. 11. | Dynasore interferes with the internalization of HIV-89.6-EGFP virus in CD4-negative and positive HeLa cells. |
| Supplementary Fig. 12. | Efficacy of RetroNectin and spinoculation HIV-1 infection techniques. |
| Supplementary Fig. 13. | Human T helper cells are positive for CD3 and CD4. |
| Supplementary Fig. 14. | Native Env-pseudotyped HIV-1 infection induces NEIs in activated T cells – a phenomenon blocked by PRR851. |
| Supplementary Fig. 15. | HIV-1 IN is found in Rab7-positive late endosomes that are segregated in one nuclear pole. |
| Supplementary Fig. 16. | Gating strategy for flow cytometry. |
| Supplementary Table 1. | Primary antibodies used for immunodetection. |
| Supplementary Table 2. | Cells used in this study. |
| Supplementary References | |

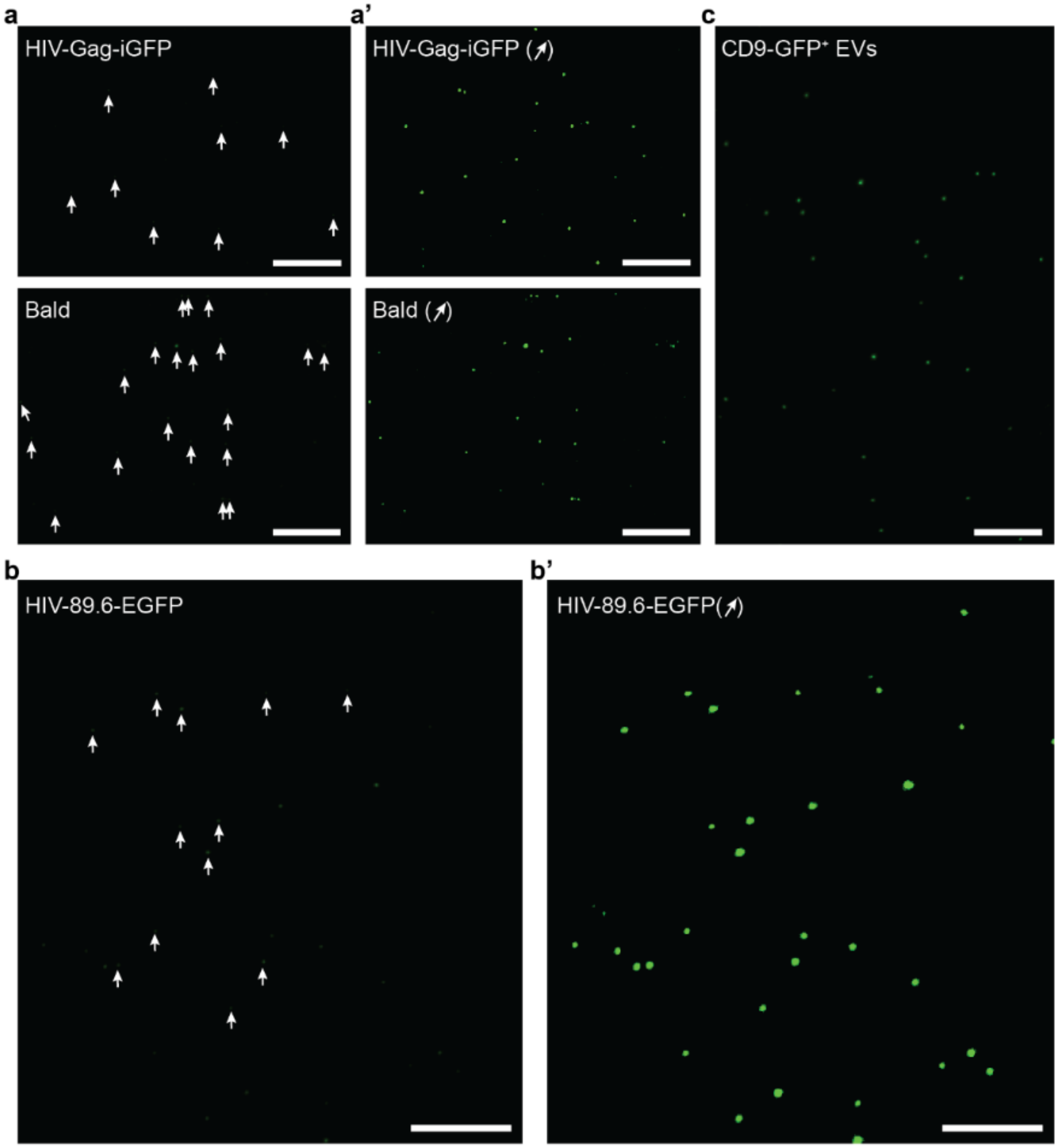


**Supplementary Fig. 1. Gag-iGFP and HIV-89.6-EGFP signals in viral particles. a-c** HIV-Gag-iGFP (a, a', top panel) and the corresponding "bald" virus, i.e. viral particles lacking the VSV-G envelope (a, a', bottom panel), HIV-89.6-EGFP containing the native envelope (b), and CD9-GFP$^{+}$ EVs derived from CD9-GFP-expressing FEMX-I cells (c) were adhered to coverslips and observed by CLSM. Arrows point to very weak (E)GFP signals in viral particles (a, b) especially compared to those of the CD9-GFP$^{+}$ EVs (c). Gag-iGFP and EGFP could be observed after image post-processing using Fiji (↗, a', b', respectively). The (E)GFP fluorescence was quantified and data are presented in the main text. Scale bars, 10 µm.

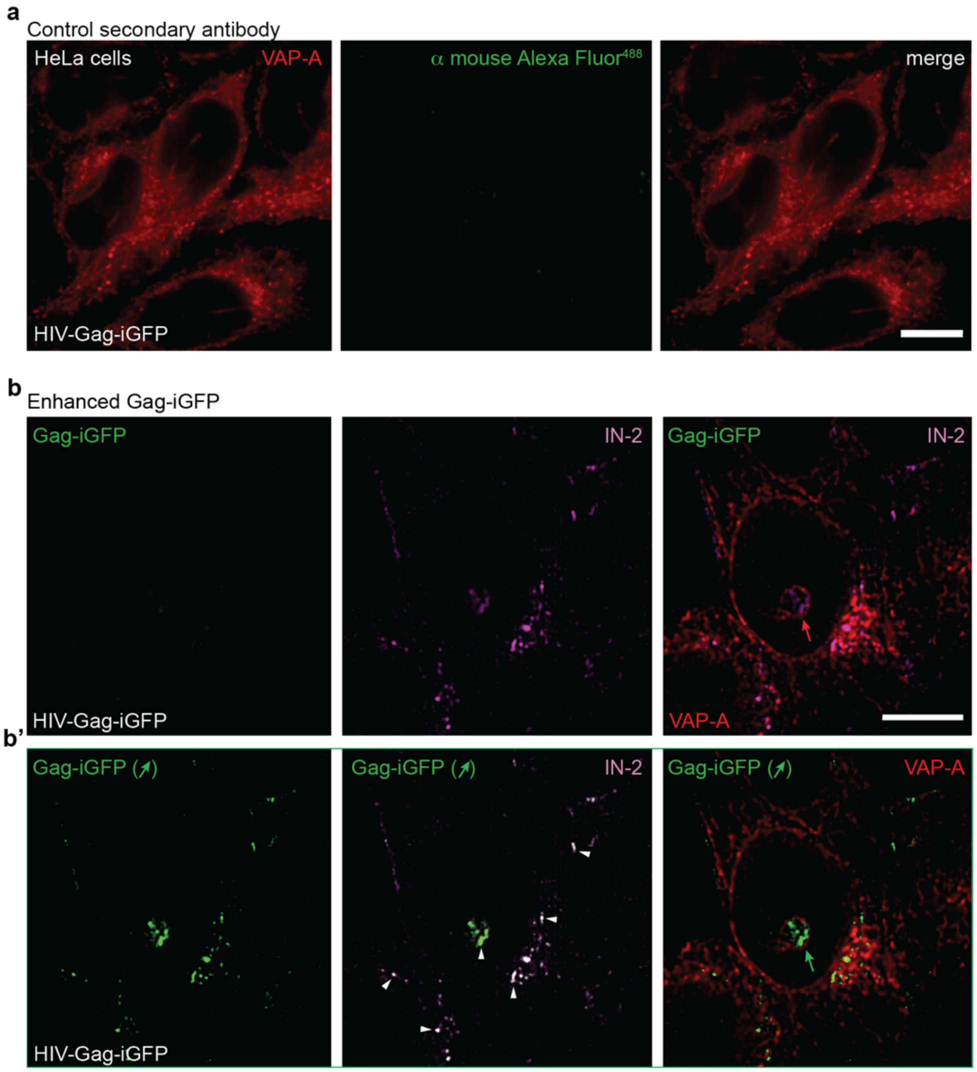


**Supplementary Fig. 2. Colocalization of HIV-1 IN and Gag-iGFP in NEI. a** HeLa cells were 1-hour infected with HIV-Gag-iGFP before immunolabeling of VAP-A followed by Alexa Fluor™ 647 (pseudo-colored in red) and Alexa Fluor™ 488 (green)-conjugated secondary antibodies; the latter was used for the detection of HIV-1 IN in all figures except panel b. Samples were observed by CLSM and single sections are displayed. Note the absence of signal when primary HIV-1 IN antibody is omitted. **b, b'** HeLa cells were treated as in panel a prior to double immunolabeling for VAP-A and HIV-1 IN. IN-2 was detected with an Alexa Fluor™ 647-conjugated anti-mouse secondary antibody (pseudo-colored in magenta). Note the absence (or low level) of Gag-iGFP under standard fluorescence acquisition (b), which could nevertheless be detected by image post-processing using Fiji (↗, b'). Red and green arrows indicate IN-2 and Gag-iGFP in the NEI, respectively, whereas white arrowheads show the colocalization of IN-2 and Gag-iGFP either in NEI or cytoplasm. In all cases, we used a MOI of 2. Scale bars, 10 µm.

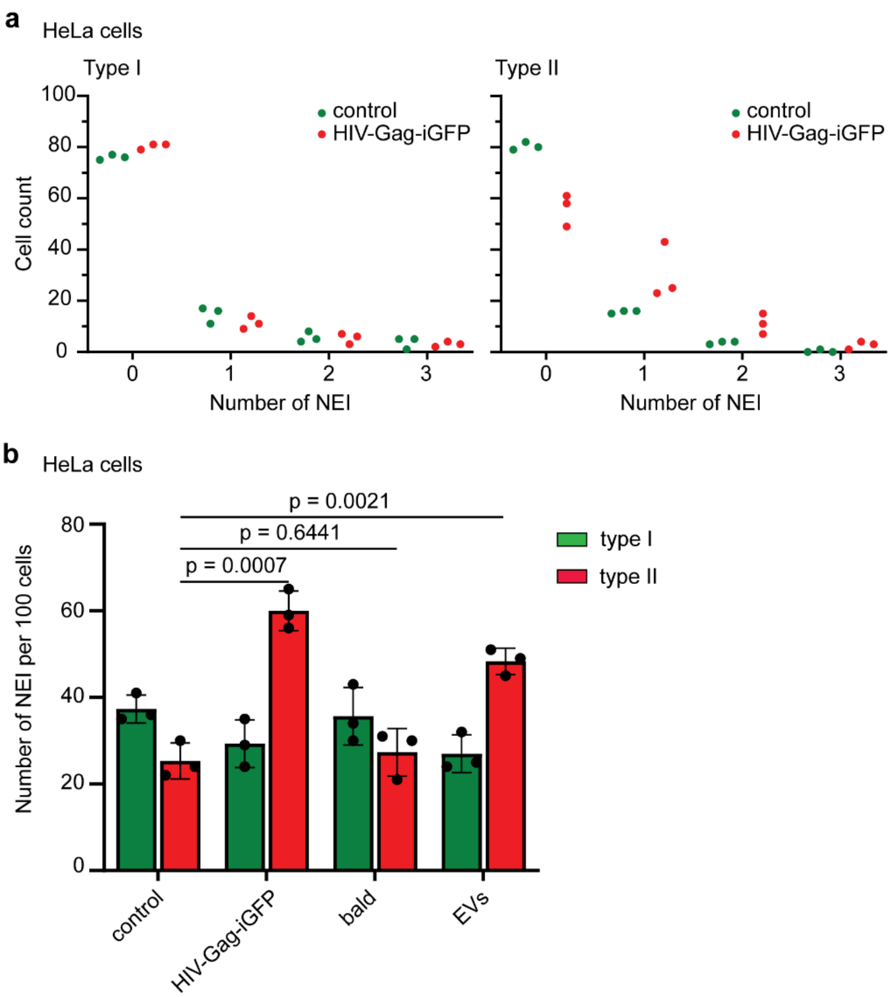


**Supplementary Fig. 3. HIV-Gag-iGFP infection increases the number of type II NEI. a, b** HeLa cells were infected either with HIV-Gag-iGFP (a, b) or "bald" (b) viruses, or alternatively incubated with EVs (final amount of 1 x $10^9$ particles) derived from FEMX-I cells (b) for 3 hours before double immunolabeling for SUN2 and VAP-A. As control, cells were neither infected nor incubated with EVs. Cells were analyzed by CLSM. The amount of type I or II NEIs per cell (a) or their distribution among cell populations (b) were quantified. The mean of individual experiments (a, b) ± S.D. (b) are shown ($n$ = 3; 100 cells per experiment). In all cases, we used a MOI of 2. $P$ values are indicated.

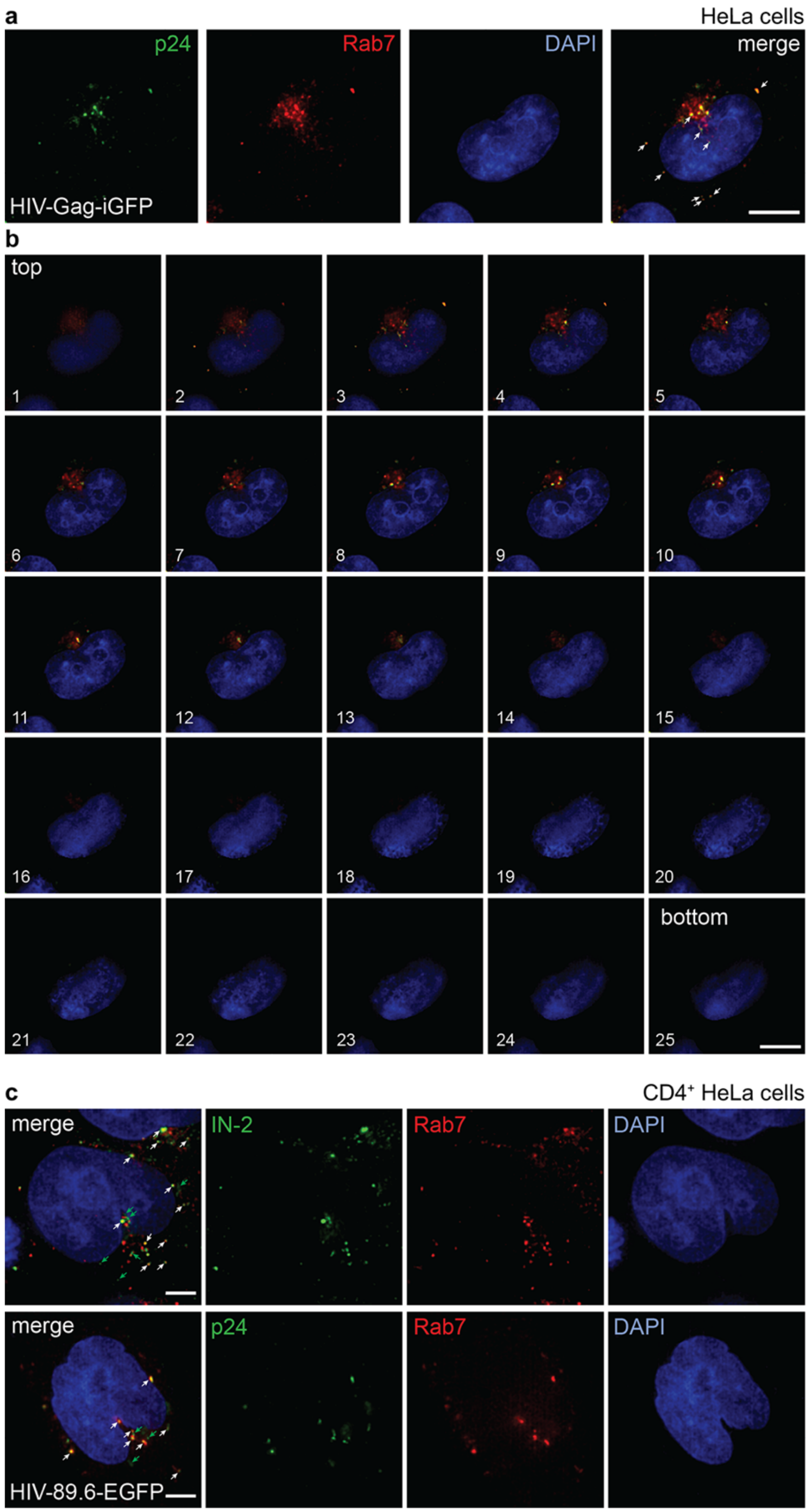


**Supplementary Fig. 4. Viral HIV-1 p24 antigen is associated with Rab7$^{+}$ late endosomes. a-c** HeLa (a, b) and CD4$^{+}$ HeLa (c) cells were 1-hour infected with HIV-Gag-

iGFP or HIV-89.6-EGFP prior to immunolabeling for HIV-1 p24 or IN-2 (green) and Rab7 (red). Nuclei were counterstained with DAPI (blue). Samples were observed by CLSM. Composite images (a, c) or 25 serial sections (0.4-μm step) from top to bottom (b) are shown. Colocalization of p24 or IN-2 and Rab7 is indicated by white arrows, while viral components alone are marked by green arrows. We used a MOI of 2. Scale bars, 5 (c) or 10 (a, b) μm.

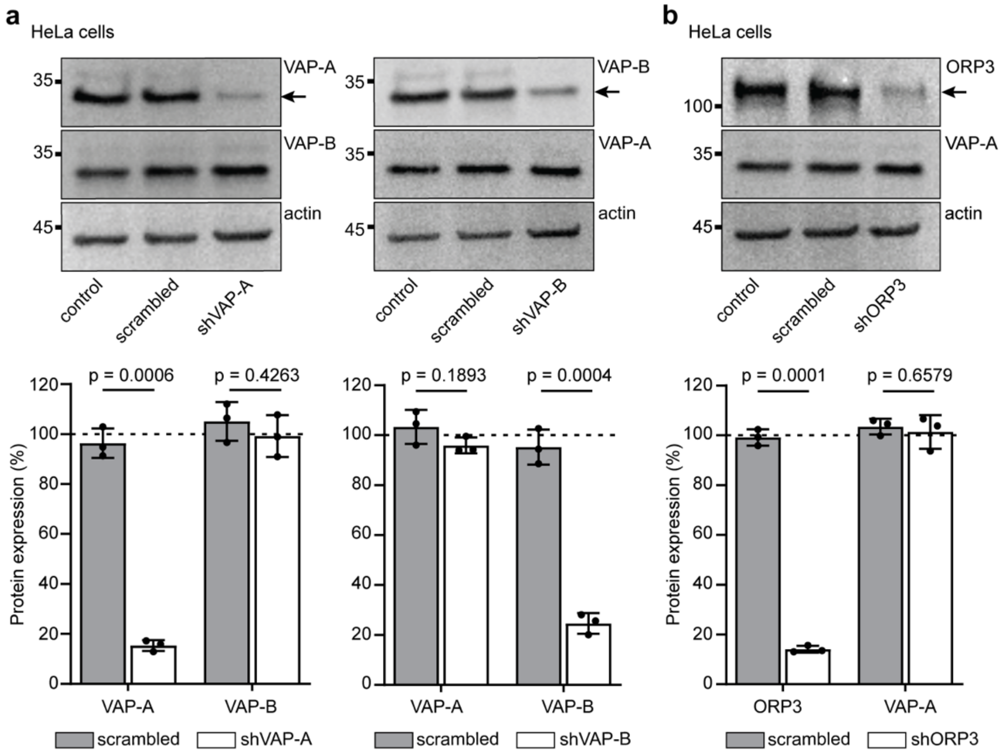


**Supplementary Fig. 5. Silencing VAP-A, VAP-B and ORP3 in HeLa cells. a, b** HeLa cells untransfected (control) or stably transfected with plasmids carrying scrambled shRNA, shVAP-A, shVAP-B, or shORP3 were analyzed by immunoblotting for VAP-A, VAP-B, ORP3, or actin. Molecular mass markers (kDa) are indicated. All samples loaded are coming from the same experiment and run in parallel. Arrows indicate the protein of interest. Representative blots are shown (a, b, upper panels). The relative expression of VAP-A/B (a, lower panels) or ORP3 (b, lower panel) was quantified by comparison with control samples (dashed lines). The samples were normalized to actin. Means ± S.D. and individual values for each experiment are shown ($n$ = 3). $P$ values are indicated.

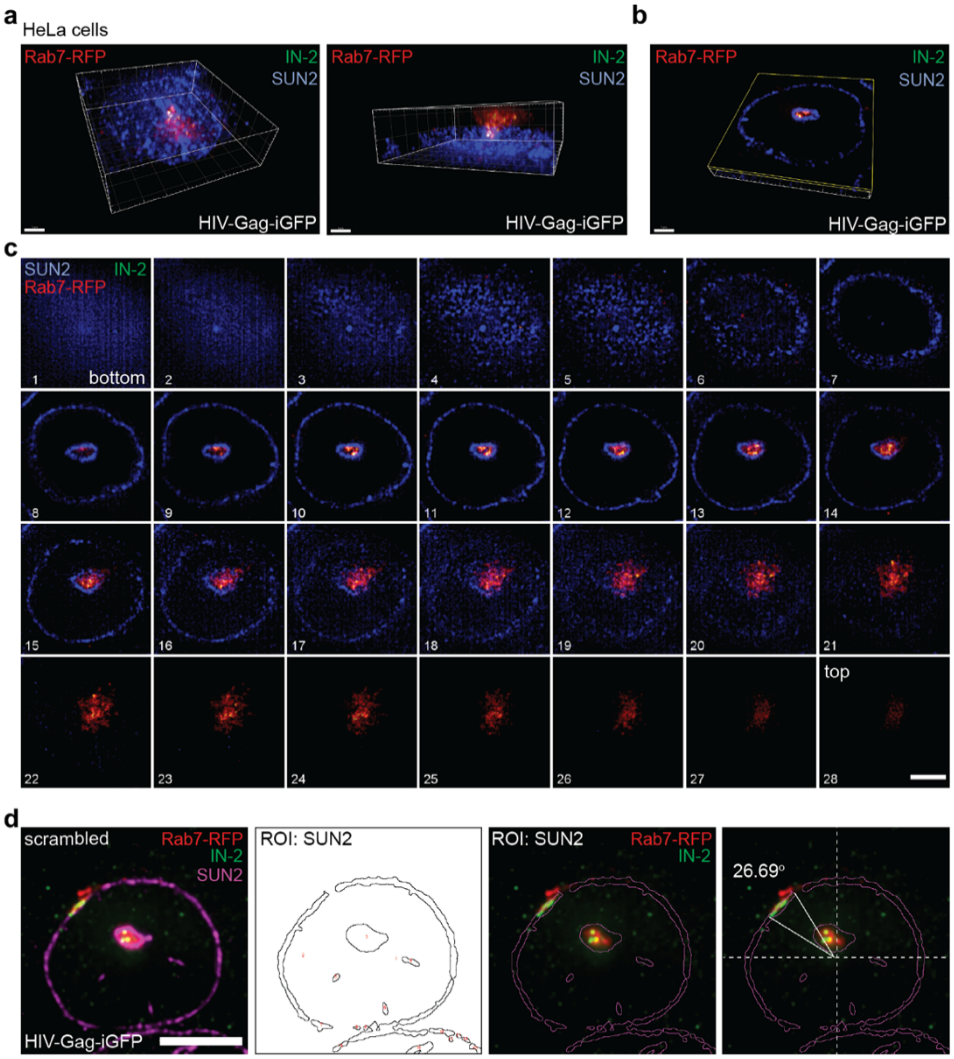


**Supplementary Fig. 6. HIV-1 integrase enters the NR *via* late endosomes with a restricted localization at the perinuclear region. a-d** Untransfected (a-c) or scrambled shRNA-transfected HeLa (d) cells expressing Rab7-RFP (red) were 1-hour infected with HIV-Gag-iGFP prior to double immunolabeling for SUN2 (blue) and HIV-1 IN (IN-2, green). Serial sections of 0.3-µm step from bottom to top of sample were acquired by CLSM, then a 3D image was rendered using Imaris software. Composite images showing the nucleus (a) and a sliced image (0.9 µm of thickness) with a transverse section of NEI (b) are shown. Each single sections from the bottom to the top of cell is presented (c), while a single section is shown (d). At the nuclear membrane, highlighted by SUN2 staining, a region of interest (ROI) was drawn and overlaid with the Rab7-RFP and IN-2 labeling. An axis was then placed in the center of the nucleus in correspondence to the region of highest IN-2 labeling and from there the angle was measured for the region in which IN-2 (and Rab7) accumulated (d). Note the presence of both IN-2 and Rab7-RFP in NEI. In all cases, we used a MOI of 2. Scale bars, 5 (a, b) or 10 (c, d) µm.

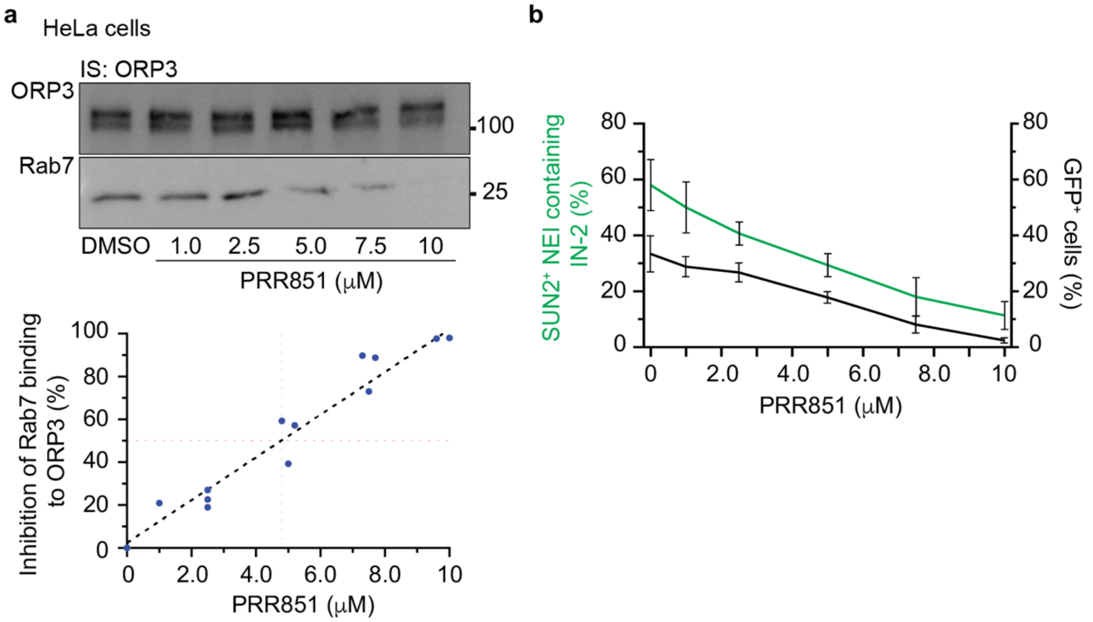


**Supplementary Fig. 7. Impact of PRR851 concentration on Rab7 binding to ORP3, entry of HIV-1 IN in NEIs and productive infection. a** HeLa cells were solubilized and detergent lysates were incubated with DMSO or various concentrations of PRR851 as indicated for 30 minutes on ice, followed by IS of ORP3. The bound fractions were probed by IB for ORP3 and Rab7 (upper panel). The percentage of inhibition of Rab7 binding to ORP3 was presented using a simple linear regression (lower panel). Molecular mass markers (kDa) are indicated. All samples loaded are coming from the same experiment and run in parallel. **b** HeLa cells were pretreated with DMSO or different concentrations of the drug as indicated for 30 minutes before infection with HIV-Gag-iGFP for 1 hour in the presence of drugs. Samples were double immunolabeled for SUN2 and HIV-1 IN (IN-2) and observed by CLSM. The number of $SUN2^+$ NEIs containing IN-2 was quantified (50 NEIs per experiment, means ± S.D. are shown, *n* = 3, left y axis). Alternatively, drug-pretreated HeLa cells were 6-hour infected followed by an additional 24 hours of incubation in the presence of PRR851, but in the absence of virus. Samples were processed by flow cytometry. DMSO-treated infected and noninfected cells were used as positive and negative controls, respectively. The percentage of $GFP^+$ cells is plotted (means ± S.D. are shown, *n* = 3, right y axis). In all cases, we used a MOI of 2.

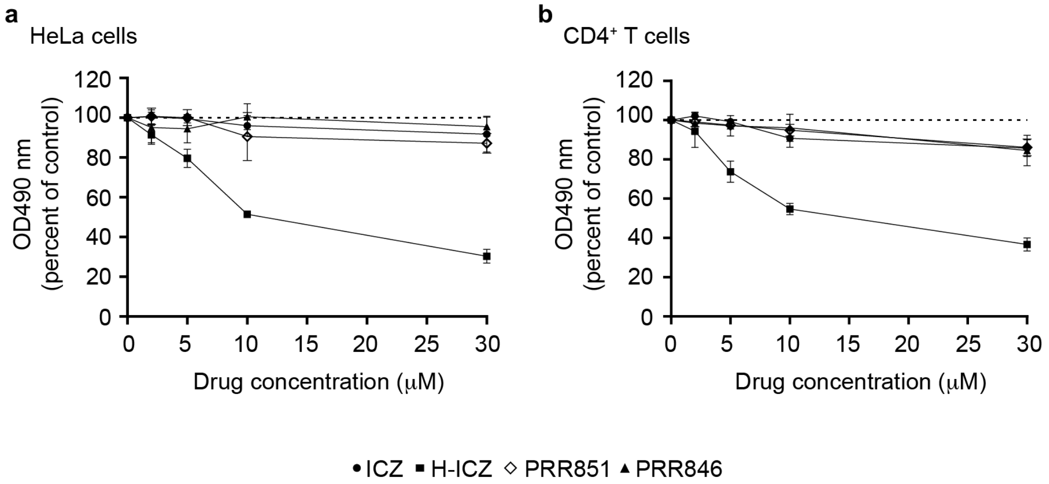


**Supplementary Fig. 8. Evaluation of drug toxicity on HeLa and primary CD4$^+$ T cells.** **a, b** Drug toxicity was evaluated on HeLa (a) and PHA/IL-2-activated CD4$^+$ T (b) cells using MTS tetrazolium assay after their incubation for 48 hours with DMSO alone (control, dashed line) or different concentrations of chemical drugs as indicated. Means ± S.D. are shown ($n$ = 3).

## Supplementary Note 1
## (Information relative to HeLa cells infected with HIV-1 virus containing native Env)

All data described so far refer to HIV-1 pseudotyped with VSV-G. To obtain information about the native virus, we employed p89.6 ΔE ΔN SF-EGFP pseudotyped with autologous HIV 89.6 Env (abbr. HIV-89.6-EGFP) containing the *bona fide* envelope glycoprotein gp160. As recipient cells, we used HeLa cells ectopically expressing CD4. Before infection, they were pretreated with ICZ and PRR analogues, and then 6-hour infected using a RetroNectin-based infection method (see Methods). Forty-eight hours later, EGFP was observed in both cytoplasm and nucleus [1] of DMSO and PRR846-treated cells using ICC (Supplementary Fig. 9a). Virtually no EGFP was detected in ICZ- and PRR851-treated cells. Flow cytometry data confirmed the significant reduction of the number of EGFP$^+$ cells (Supplementary Fig. 9b, c). Interestingly, when the same experiment was performed with native HeLa cells as recipients, EGFP expression was greatly reduced, but not totally abolished (Supplementary Fig. 10a, b, see below). The few remaining CD4-negative HeLa cells that expressed EGFP were also sensitive to PRR851 (Supplementary Fig. 10a, b), suggesting that the VOR complex is essential for nuclear transfer of the viral components, regardless of the mechanism of viral particle internalization, which may be CD4-dependent

or CD4-independent (see below). The impact of cell surface CD4 in the cellular internalization of HIV-89.6-EGFP was also highlighted by ICC, as evidenced by the significant reduction of the total IN-2 or p24 in the cytoplasm of native HeLa compared to $CD4^+$ HeLa cells after one hour of infection (Supplementary Fig. 10c, d). The total numbers of HeLa cells containing IN-2 or p24 were also significantly reduced in the absence of CD4 (Supplementary Fig. 10e). Of note, PRR851 did not interfere with internalization of viral particle components (Supplementary Fig. 10d, e), consistent with the data presented in the main text (Fig. 4d-h). Although the amount of IN-2 and p24 was limited in the cytoplasm of infected CD4-negative HeLa cells, both viral components reached the VAP-$A^+$ NEIs (Supplementary Fig. 10f, g), which could explain the reduced, but still present, EGFP level in native HeLa cells (see below). The number of NEIs also increased in infected $CD4^+$ HeLa cells (33.3 ± 4.2 NEIs per 50 cells, $n = 3$) compared to native HeLa cells (18.0 ± 3.0 NEIs per 50 cells), the latter being at the level of native condition, i.e. without infection (see Supplementary Fig. 3). As shown above with VSV-G-pseudotyped HIV-1 (Fig. 4d-g), PRR851 had a negative impact on the entry of viral components into VAP-$A^+$ NEIs (Supplementary Fig. 10f, g), consistent with low EGFP levels upon incubation with the drug.

Endocytosis appears important in the internalization of the viral particles as the pretreatment of native HeLa cells for 30 minutes with DNS, as described in the main text, completely impeded the presence of intracellular IN-2 after 3-hour infection with HIV-89.6-EGFP (Supplementary Fig. 11a, c). The total IN-2 was also severely reduced in $CD4^+$ HeLa cells after the same treatment (Supplementary Fig. 11b, c). Nonetheless, a small fraction of HIV-1 IN escaped DNS interference in some cells, especially when a higher concentration of virus was applied (Supplementary Fig. 11d-f) (see below and Discussion). In line with previous data with HIV-Gag-iGFP (Supplementary Fig. 4a, b), after 1-hour infection, internalized IN-2 and p24 were associated with $Rab7^+$ late endosomes, including those found in NEIs of $CD4^+$ HeLa cells (Pearson's R correlation coefficients: 0.81 ± 0.09 and 0.79 ± 0.09 for IN-2 or p24 with Rab7, respectively, $n = 10$ cells, $p < 0.01$) (Supplementary Fig. 4c). It should be noted that viral proteins were also found outside the $Rab7^+$ endosomal structures, suggesting that they escape degradation in the endo-lysosomes (Supplementary Fig. 4c). Of note, the internalization process monitored by IN-2 was also impaired in the absence of the native envelope component (*Env* gene) as demonstrated with "bald" virus, irrespective of the amount of virus used (MOI ranging from 2 to 8) (Supplementary Fig. 11g, h).

As mentioned above, we surprisingly observed a small but present population of infected native HeLa cells expressing EGFP (Supplementary Fig. 10). Although the dependence of the HIV-1 productive infection on CD4 is well established [2], the attachment of viral particles to HeLa cells could involve other lectin-like cell surface factors [3-5], which might favor a CD4-independent endocytosis process leading to the expression of the

reporter gene *EGFP* in a limited number of cells. This may be particularly true with the current method used to increase the level of infection. i.e. RetroNectin, which is based on a recombinant fibronectin fragment that binds to cells via its interaction with cell surface integrin receptors. Therefore, in the absence of CD4, a tiny fraction of the RetroNectin-virus complex present in the vicinity of the cell membrane can penetrate by other non-specific mechanisms of endocytosis, e.g., macropinocytosis, and thus produce a small, but present expression of the reporter gene *EGFP*. This technique has already been used to overcome limitations associated with the resistance of $CD34^+$ hematopoietic stem/progenitor cells to HIV infection in vitro [6]. These $CD34^+$ cells have a low level of CD4 expression. This prompted us to investigate this issue using an alternative method of infection, namely spinoculation [7]. This method concentrates the viral particles on the cell surface, among other notable biochemical/physical effects on the cells [8], by a step of gentle centrifugation at 4°C of the cells of interest with the viral particles. This technique is widely used in the HIV field. Interestingly, infection of $CD4^+$ HeLa cells with HIV-89.6-EGFP by spinoculation gave the same results as the RetroNectin technique, with the EGFP expression impacted by the presence of PRR851 (Supplementary Fig. 12a). In contrast, in spinoculation-infected native HeLa cells the number of $EGFP^+$ cells was significantly reduced to 3.12 ± 2.35%, compared with 6.24 ± 3.94% obtained with RetroNectin (Supplementary Fig. 12a). Because spinoculation yielded few $EGFP^+$ cells, the addition of PRR851 did not significantly reduce their number compared with the control (1.28 ± 0.98% vs. 3.12 ± 2.35%, respectively), whereas this was the case when RetroNectin was used (Supplementary Fig. 12a). It remains to be determined whether and how the internalization of RetroNectin complex can promote the fusion of virus particles with the endosomal membrane in the absence of CD4, and hence, escape the degradation into the lysosomes. These phenomena did not occur with spinoculation.

In addition to the impact of the infection methods, we also assessed the expression of the structural protein Gag. In our construct (i.e. p89.6 ΔE ΔN-SF-EGFP), which is used with native Env, the *EGFP* reporter gene is not controlled by the HIV-1-LTR promoter, but depends on the internal promoter of the spleen focus-forming virus (see Methods). As noted by Carter and colleagues, a difference was previously observed between EGFP and Gag expression, which could be explained by early expression of EGFP, i.e., before the expression of HIV-1-LTR-driven genes [9]. This was then addressed by monitoring EGFP and Gag expression by flow cytometry. The latter was detected using an anti-Gag antibody. Using the RetroNectin-based method, the number of double $EGFP^+Gag^+$ cells (i.e. 2.36 ± 0.22%) decreased significantly compared to the overall EGFP expression (6.85 ± 0.31%) in CD4-deficient HeLa cells, indicating that productive infection of the gene under the HIV-LTR promoter still occurs, but at a very low level (Supplementary Fig. 12b). This small amount of double-positive cells is still responsive to drugs, as it was reduced to less than 1% (Supplementary Fig. 12b). In contrast, double $EGFP^+Gag^+$ cells were almost

absent, or even at background level (i.e. 1.44 ± 0.42%), in cells infected by spinoculation, and thus were not affected by the addition of the drug (Supplementary Fig. 12c). Hence, infection methods can influence the productive infection of CD4-depleted HeLa cells, wherein slight differences are observed when monitoring an endogenous viral gene versus a reporter gene, consistent with previous study [9].

Altogether, ICZ and PRR851 blocked the nuclear transfer of HIV-1 components via NEIs and productive infection, regardless of the nature of env components (i.e. VSV-G or *env* gene products) present in the viral particles in heterologous cellular system such as HeLa cells expressing or not CD4. Infection methods also have a slight impact on productive infection in heterologous cells lacking CD4.

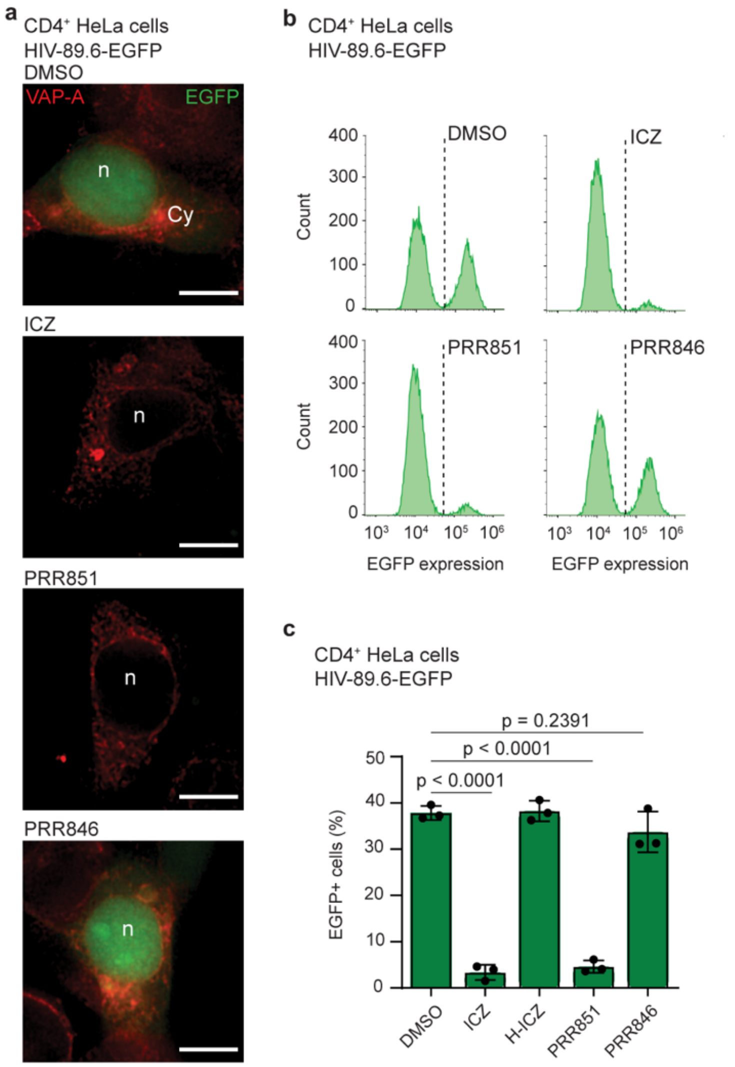


**Supplementary Fig. 9. The VOR complex inhibition by itraconazole or PRR851 impedes the HIV-1-associated EGFP expression. a-c** $CD4^+$ HeLa were pretreated with 10 µM ICZ, PRR851, PRR846, H-ICZ or DMSO for 30 minutes before HIV-89.6-EGFP infection for 6 hours, after which they were washed and incubated for 48 hours in the presence of drugs. Samples were processed either for ICC (a) or flow cytometry (b, c). For ICC, fixed cells were immunolabeled for VAP-A and observed by CLSM, while they were trypsinized (in the case of HeLa cells) for flow cytometry. Representative fluorescent images (a) and flow histograms (b) are displayed. DMSO-treated, noninfected cells were used for the gating. (b). The percentage of $EGFP^+$ cells is plotted (c). Means ± S.D. and individual values for each experiment are shown ($n$ = 3). Cy, cytoplasm; n, nucleus. In all cases, we used a MOI of 2. $P$ values are indicated. Scale bars, 10 µm.

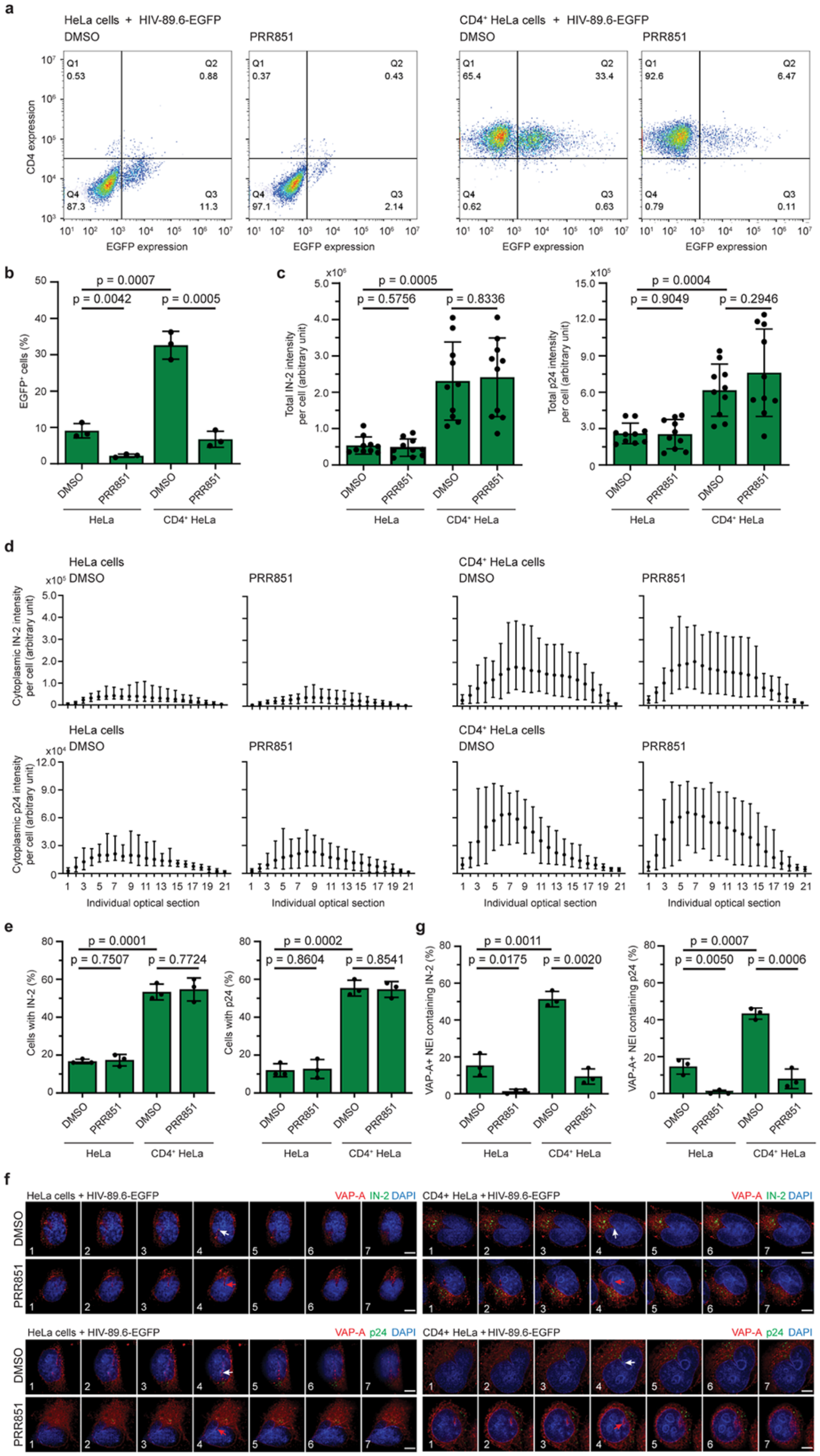


**Supplementary Fig. 10. Cell surface CD4 stimulates the uptake of HIV-1 components in HeLa cells. a, b** HeLa or CD4$^{+}$ HeLa cells were pretreated with DMSO or 10 μM

PRR851 for 30 minutes before 6-hour HIV-89.6-EGFP infection in the presence of drug, after which they were incubated for additional 48 hours in the absence of virus and drugs. Samples were labeled using fluorochrome-conjugated anti-CD4 antibodies and analyzed by flow cytometry. They were assessed for CD4 and EGFP expression by placing a quadrant (Q) gate on the negative sample (i.e. DMSO-treated, noninfected HeLa or $CD4^+$ HeLa cells without CD4 antibody) (Q4). Percentages of cells are shown for each quadrant (a), and total $EGFP^+$ cells per condition (b, *n* = 3). **c-g** HeLa and $CD4^+$ HeLa cells pretreated as above were 1-hour infected in the presence of drug. Cells were fixed prior to double immunolabeling for VAP-A (red) and HIV-1 IN (IN-2) or p24 (green). Nuclei were counterstained with DAPI (blue). Twenty-one serial sections of 0.3-µm step from bottom to top of sample were acquired by CLSM. The total fluorescence intensity of IN-2 or p24 in the cytoplasm was measured as well as for each individual section (c, d, *n* = 10 cells per condition), where the means with the range of fluorescence signal are displayed. The number of cells that are positive for IN-2 or p24 was quantified and the mean ± S.D. are shown (e, 50 cells per experiment, *n* = 3). Seven consecutive sections encompassing NEI of representative cells are displayed (f). White and red arrows indicate the presence or absence of IN-2 (or p24) in the NEIs, respectively. The number of VAP-$A^+$ NEIs that contain either IN-2 or p24 was quantified (g). At least 50 NEIs were evaluated per experiment (*n* = 3). In all cases, the means ± S.D. and, where appropriate, the individual values for each cell or experiment are indicated. We used a MOI of 2. *P* values are indicated. Scale bars, 5 µm.

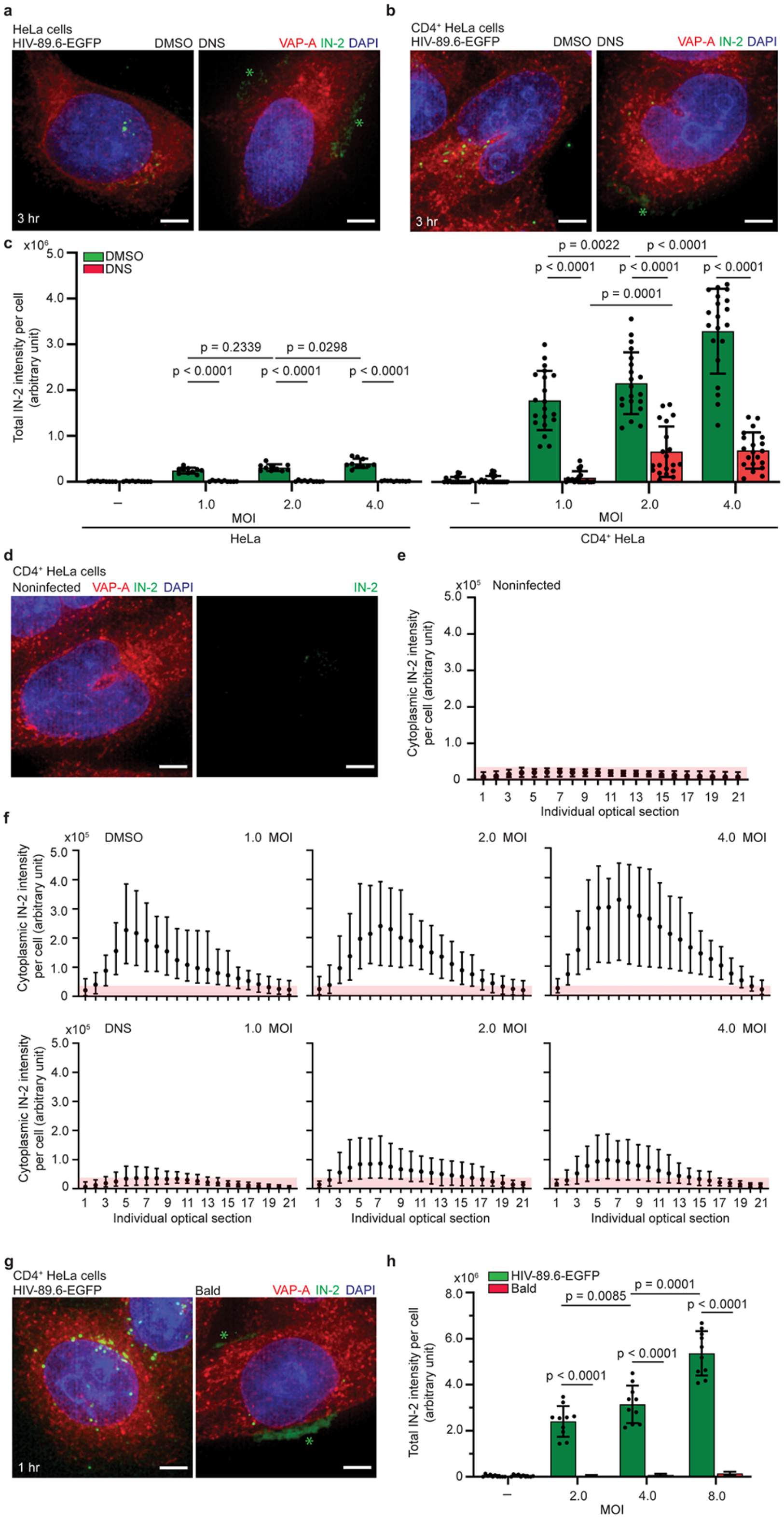


**Supplementary Fig. 11. Dynasore interferes with the internalization of HIV-89.6-EGFP virus in CD4-negative and positive HeLa cells. a-f** HeLa (a, c) and CD4$^{+}$ HeLa (b-f) cells were pretreated with DMSO or 80 µM DNS for 30 minutes before 3-hour HIV-

89.6-EGFP infection. As negative control, $CD4^+$ HeLa cells were noninfected (d). Cells were fixed prior to double immunolabeling for VAP-A (red) and IN-2 (green). Nuclei were counterstained with DAPI (blue). Twenty-one serial sections of 0.3-μm step from bottom to top of sample were acquired by CLSM. Composite images are shown (a, b, d). The green channel of noninfected cells (d) that was used to evaluate the fluorescent background (e, pink area) is displayed. Total IN-2 intensity per cell was quantified (c) and the mean ± S.D. are shown ($n$ = 10 (HeLa) and 20 ($CD4^+$ HeLa) cells per condition). The fluorescence intensity of IN-2 in the cytoplasm was measured for each section, where the means ± S.D indicating the range of fluorescence signal are displayed (e, f, $n$ = 20 cells per condition). **g, h** $CD4^+$ HeLa cells were 1-hour infected with HIV-89.6-EGFP or corresponding “bald” virus and then analyzed as above. Composite images (g) and total IN-2 intensity per cell (h) are displayed. Means ± S.D. and individual values for each cell are indicated ($n$ = 10 cells per condition). Green asterisks indicate viral particles at the cell membrane (a, b, g). We used a MOI of 1 (c), 2 (a-f, h), 4 (c, h) or 8 (g, h) or equivalent in the case of the “bald” virus. $P$ values are indicated. Scale bars, 5 μm.

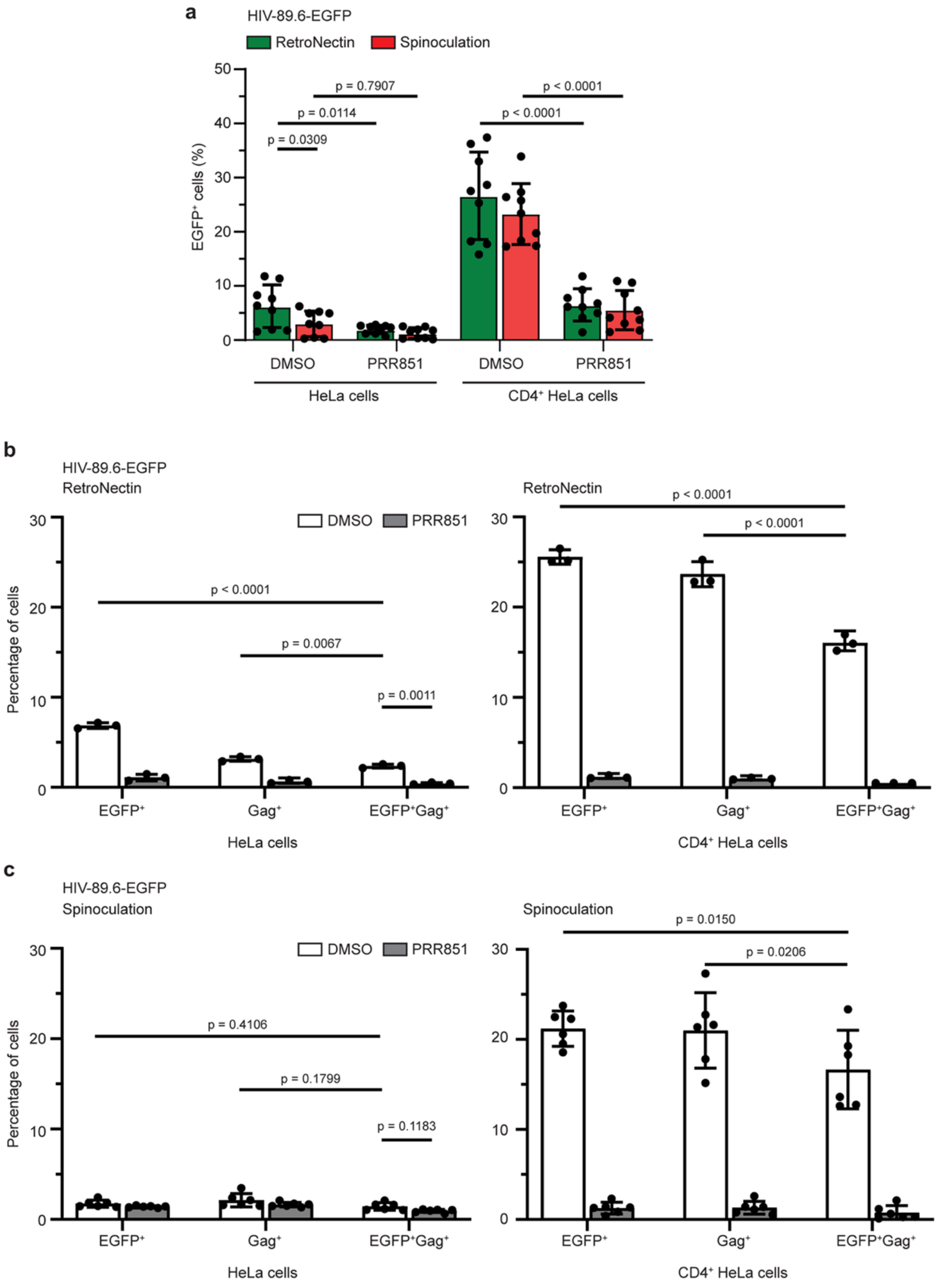


**Supplementary Fig. 12. Efficacy of RetroNectin and spinoculation HIV-1 infection techniques. a-c** HeLa and CD4+ HeLa were pretreated with DMSO or 10 µM PRR851 for 30 minutes, then 6-hour infected with HIV-89.6-EGFP using two different techniques: RetroNectin (a, b) or spinoculation (a, c). In both cases, media were replaced prior to 48-hour incubation at 37°C in the presence of drugs. Trypsinized cells were either analyzed immediately (a) or fixed, permeabilized, and labeled using fluorochrome-conjugated anti-

Gag antibodies (b, c) prior to flow cytometry analysis of EGFP and/or Gag expression. For the gating strategy, the viral infection was omitted. Total $EGFP^+$ (a-c) or $Gag^+$ and double-positive ($EGFP^+Gag^+$) (b, c) cells are displayed. Means ± S.D. and individual values for each experiment ($n$ = 9 (a), 3 (b), or 6 (c)) are shown. In all cases, we used a MOI of 2. $P$ values are indicated.

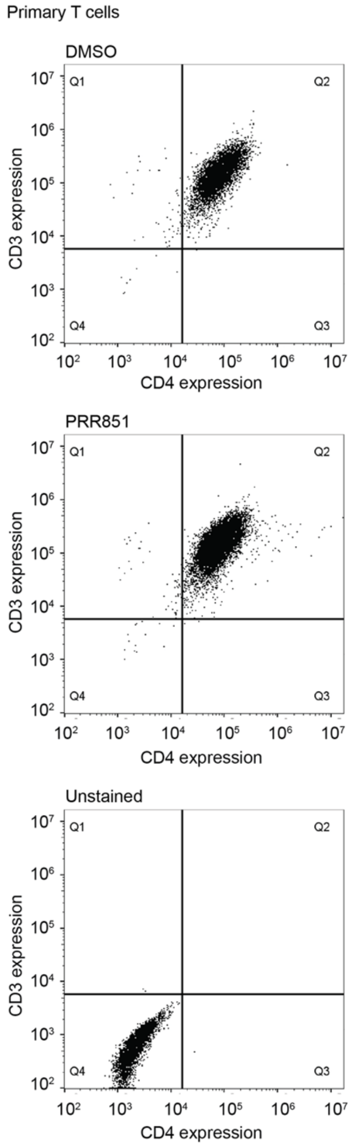


**Supplementary Fig. 13. Human T helper cells are positive for CD3 and CD4**. PHA/IL-2-activated $CD4^+$ T cells were 30-minute pretreated with 10 µM ICZ, PRR851, or PRR846, or equivalent volume of DMSO before 6-hour HIV-89.6-EGFP infection, washed, and incubated for 48 hours in the presence of drugs. Cells were labeled using fluorochrome-conjugated anti-CD3 and CD4 antibodies for 45 minutes on ice, then analyzed by flow cytometry. As negative control, antibody staining was omitted (unstained). Cells were assessed for CD3/CD4 expression by placing a quadrant gate where the unstained sample is positioned in quadrant Q4. The double-positive population (Q2) was then evaluated for productive infection of the virus by measuring EGFP expression (see Fig. 5a, b). In all cases, we used a MOI of 2.

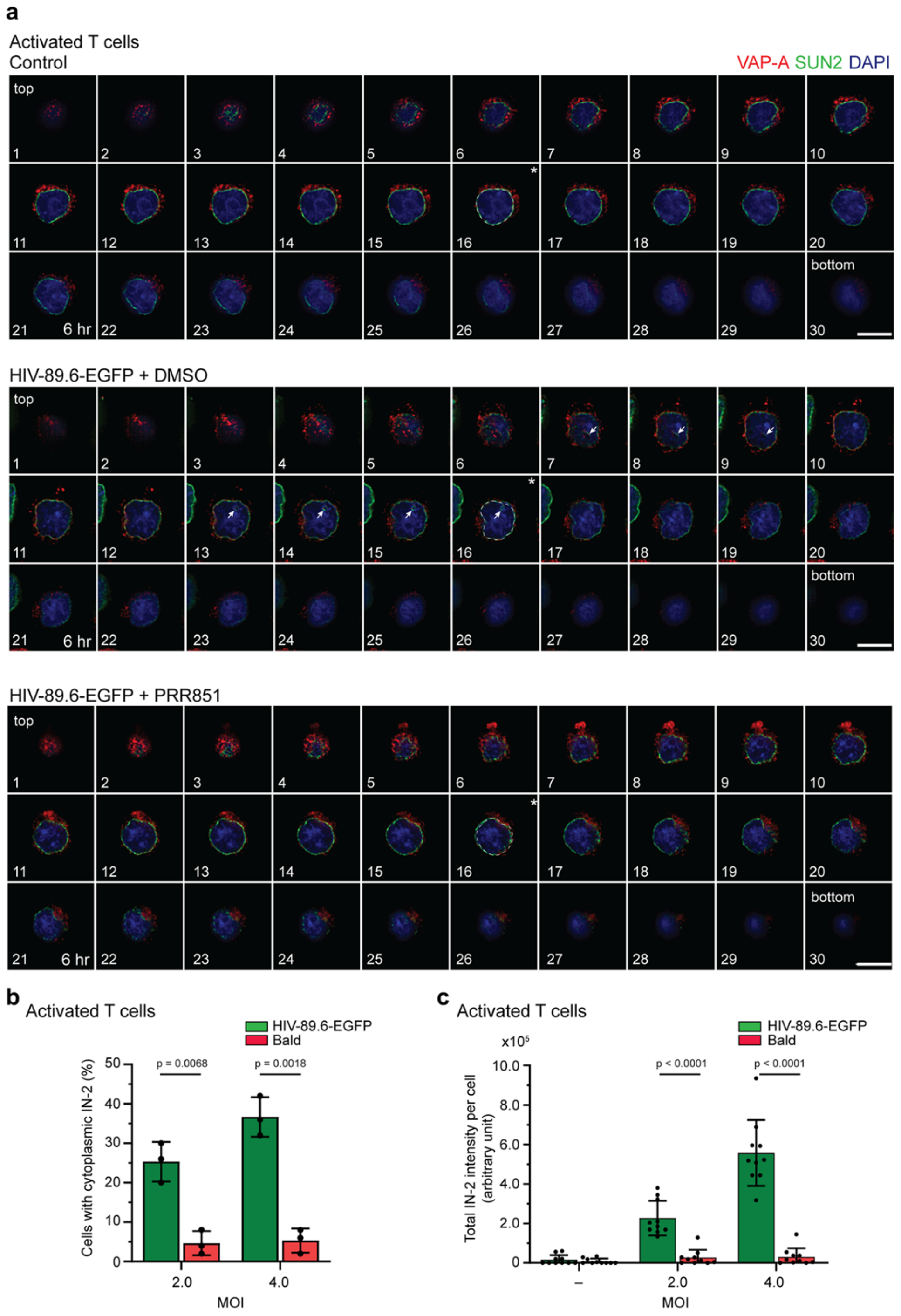


**Supplementary Fig. 14. Native Env-pseudotyped HIV-1 infection induces NEIs in activated T cells – a phenomenon blocked by PRR851. a** PHA/IL-2-activated primary CD4$^{+}$ T cells were noninfected (control) or infected with HIV-89.6-EGFP for 6 hours. In the latter case, cells were pretreated with DMSO or 10 μM PRR851 for 30 minutes before infection. Drug remained present during the infection. Cells were processed for ICC by double immunolabeling for VAP-A (red) and SUN2 (green). Nuclei were counterstained

with DAPI (blue). Thirty serial sections of 0.3-µm step from top to bottom of representative cells were acquired by CLSM. Note the presence of NEIs in the infected cell (arrows), which are abolished after PRR851 treatment. Dashed lines indicate the general organization of the nuclear membrane that is altered in infected cells (see panel marked with an asterisk). **b, c** PHA/IL-2-activated $CD4^+$ T cells were 3-hour infected with HIV-89.6-EGFP or corresponding "bald" virus prior to double immunolabeling for VAP-A and HIV-1 IN (IN-2). Samples were observed by CLSM (see Fig. 6i, j). Percentage of cells with cytoplasmic IN-2 were quantified (b). Means ± S.D. and individual mean values of each experiment are indicated (≥ 50 cells per condition, $n = 3$). The total fluorescence intensity of IN-2 in the cytoplasm was measured using serial 21 sections through a cell using the cytoplasmic compartments as a region of interest (c). Means ± S.D. and individual values for each cell are indicated ($n = 10$ cells per condition). In all cases, we used a MOI of 2 or equivalent in the case of the "bald" virus. *P* values are indicated. Scale bars, 5 µm.

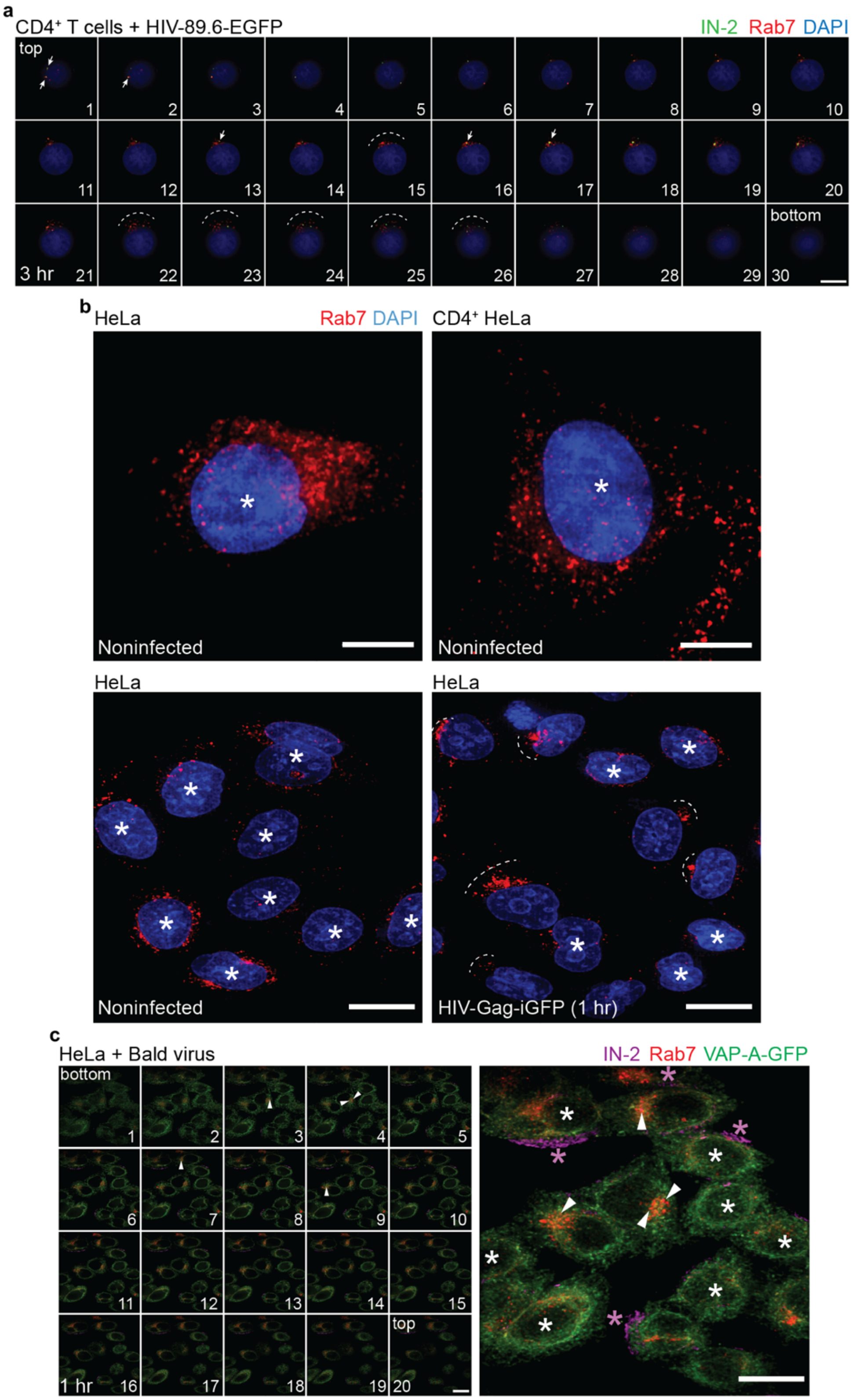


**Supplementary Fig. 15. HIV-1 IN is found in Rab7-positive late endosomes that are segregated in one nuclear pole. a** PHA/IL-2-activated primary CD4$^+$ T cells were 3-hour

infected with HIV-89.6-EGFP before double immunolabeling for HIV-1 IN (IN-2, green) and Rab7 (red). Nuclei were counterstained with DAPI (blue). The samples were observed by CLSM. Serial sections of 0.3-μm step of a representative cell are displayed. Colocalization of IN-2 and Rab7 is indicated with arrows, while dashed lines indicate Rab7 aggregation at one pole of the nucleus. **b** Noninfected HeLa and $CD4^+$ HeLa cells or those 1-hour infected with HIV-Gag-iGFP were immunolabeled for Rab7 (red) and counterstained with DAPI (blue). Note the random distribution of $Rab7^+$ late endosomes around the nucleus in noninfected cells (white asterisks), which contrasts with those infected (dashed line; see also Supplementary Fig. 6a). **c** VAP-A-GFP-expressing HeLa cells were 1-hour infected with VSV-G-derived "bald" virus prior to double immunolabeling for Rab7 (red) and HIV-1 IN (magenta). Single sections (left panels) and a composite image (right panel) are shown. Again, a random distribution of Rab7 around the nucleus is observed in cells lacking IN-2, while those containing a tiny fraction of IN-2 in the cytoplasmic compartment (arrowhead) show partial Rab7 polarization. Most IN-2 appears outside the cells (magenta asterisks). In all cases, we used a MOI of 2 or equivalent in the case of the "bald" virus. Scale bars, 5 (a) or 10 (b, c) μm.

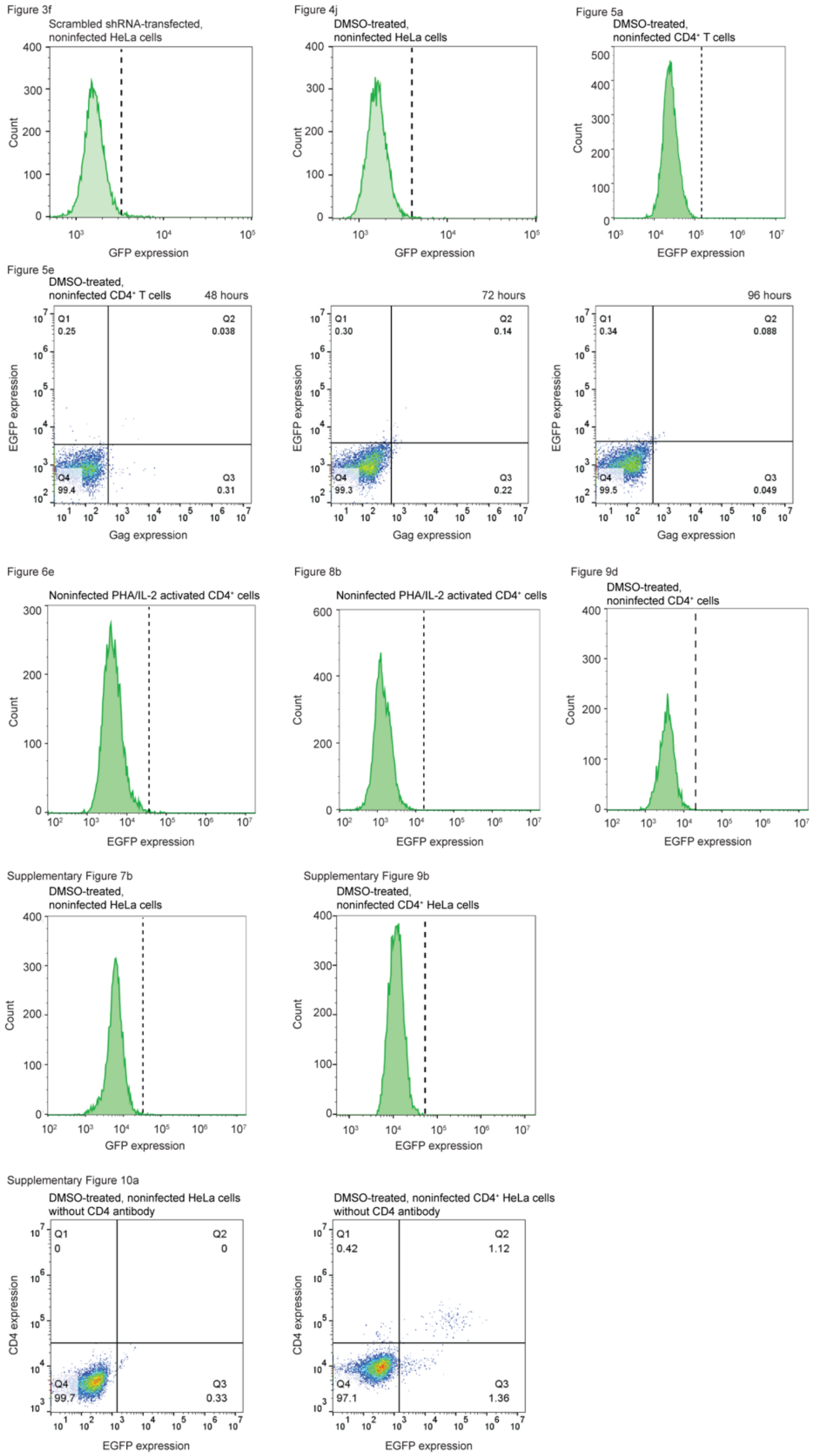


**Supplementary Fig. 16. Gating strategy for flow cytometry.** All negative controls for flow cytometry analyses used for the gating strategy are indicated. Figure numbers and cells of interest where data are presented in the main text or Supplementary Information are indicated.

**Supplementary Table 1.** Primary antibodies used for immunodetection

| Antigen | Clone/AS (host species[§]) | Manufacturer | Dilution | Representative publications (PMID) | Validation in this study |
|---|---|---|---|---|---|
| HIV-1 IN | IN-2 (m) | Santa Cruz Biotechnology | 1:50 (*ICC)<br>1:200 (IB) | 30804369<br>25808736 | Fig. 1a, b, g, j, k, l<br>Fig. 2a, b, c, g<br>Fig. 3a<br>Fig. 4c, d, g<br>Fig. 6i, k<br>Fig. 8d, e<br>Suppl. Fig. 2b<br>Suppl. Fig. 4c<br>Suppl. Fig. 6<br>Suppl. Fig. 10f<br>Suppl. Fig. 11a, b, d, e<br>Suppl. Fig. 15a, c |
| HIV-1 Gag-RDI | clone KC57 (m) | Beckman Coulter | 1:20 (FC) | 20208541 | Fig. 5e<br>Suppl. Fig. 12b, c<br>Suppl. Fig. 16 |
| HIV-1 p24 | 5 (m) | Abcam | 1:100 (ICC) | 36062074 | Suppl. Fig. 4<br>Suppl. Fig. 10f |
| VAP-A | AS A304-366A (r) | Bethyl Laboratories | 1:100 (ICC)<br>1:1000 (IB) | 34429859 | Fig. 1a, b, e, g<br>Fig. 3a, e<br>Fig. 4b, c, i<br>Fig. 6a, c, g, i<br>Fig. 7a, b<br>Fig. 8a, h<br>Fig. 9e<br>Suppl. Fig. 2a<br>Suppl. Fig. 5<br>Suppl. Fig. 9a<br>Suppl. Fig. 10f<br>Suppl. Fig. 11a, b, d, g<br>Suppl. Fig. 14a |
| VAP-B | AS A302-894A (r) | Bethyl Laboratories | 1:100 (ICC)<br>1:1000 (IB) | 34429859 | Fig. 3a, e<br>Suppl. Fig. 5a |
| ORP3 | D-12 (m) | Santa Cruz Biotechnology | 1:50 (ICC)<br>1:200 (IS) | 34429859 | Fig. 7a, b<br>Fig. 8h<br>Fig. 9e<br>Suppl. Fig. 5b<br>Suppl. Fig. 7a |

| | | | | | |
|---|---|---|---|---|---|
| | | | 1:500 (IB) | | |
| | AS A304-557A (r) | Bethyl Laboratories | 1:1000 (IB) | 34429859 | Fig. 3a, e<br>Fig. 4b<br>Fig. 7a, b<br>Fig. 8h, j<br>Fig. 9e<br>Suppl. Fig. 7a |
| Rab7 | EPR7589 (r) | Abcam | 1:50 (ICC)<br><br>1:500 (IB) | 31085713 | Fig. 2a, d, e, g<br>Fig. 4b<br>Fig. 6k, l<br>Fig. 7a, b<br>Fig. 8d, e, h<br>Fig. 9e<br>Suppl. Fig. 4a<br>Suppl. Fig. 7a<br>Suppl. Fig. 15 |
| Actin | C-2 (m) | Santa Cruz Biotechnology | 1:1000 (IB) | 36003783 | Suppl. Fig. 5 |
| CD3-PE | HIT3a (m) | Thermo Fisher Scientific | 1:20 (FC) | 32508837 | Suppl. Fig. 13 |
| CD4-BV421 | OKT4 (m) | BioLegend | 1:20 (FC) | 32330454 | Suppl. Fig. 10a<br>Suppl. Fig. 13<br>Suppl. Fig. 16 |
| CD63 | EPR22458-280 (r) | Abcam | 1:100 (ICC) | 34332599 | Fig. 2b |
| GAPDH | A1 (r) | Novus Biologicals | 1:1000 (IB) | 27602114 | Fig. 4g |
| Lamin B1 | 119D5-F1 (m) | Abcam | 1:100 (ICC)<br><br>1:1000 (IB) | 33767161 | Fig. 4g<br>Fig. 6f |
| LAMP1 | H4A3 (m) | Abcam | 1:100 (ICC) | 33731717 | Fig. 2d, e<br>Fig. 6l |
| | EPR24395-31 (r) | Abcam | 1:100 (ICC) | 35984564 | Fig. 2c |
| SUN2 | A-10 (m) | Santa Cruz Biotechnology | 1:50 (ICC) | 30982221 | Fig. 1e<br>Fig. 3e<br>Fig. 6a, c, g, l<br>Suppl. Fig. 14a |
| | ARC2311(r) | Thermo Fisher Scientific | 1:100 (ICC) | 32908309 | Fig. 3a<br>Fig. 4d<br>Fig. 6f, l |

| | | | | | |
|---|---|---|---|---|---|
| | | | | | Suppl. Fig. 6 |

§m, mouse; r, rabbit
*ICC, immunocytochemistry; IS, immunoisolation; IB, immunoblotting; FC, flow cytometry

**Supplementary Table 2.** Cells used in this study

| Cells | Origin | Source | Cell Media† | Validation (PMID) |
|---|---|---|---|---|
| HeLa | Human cervical adenocarcinoma | ATCC® CCL-2™ | DMEM | 31790455 |
| $CD4^+$HeLa | Human cervical adenocarcinoma | ARP-1109 (clone 1022) NIAID, NIH | DMEM | 1920616 |
| $CD4^+$ T | Primary human PBMC | Discovery Life Sciences | RPMI | |
| $CD4^+$ T | Primary human PBMC | Isolated in one of our laboratories (see Methods) | RPMI | |
| 293T | Human embryonic kidney cell line | ATCC® CRL-3216™ | DMEM | 25187545 |
| FEMX-I | Human melanoma | Lymph node from patient with malignant melanoma | RPMI | 23767874 28129640 30018135 |

†All cell media were supplemented with 10% fetal bovine serum, 2 mM L-glutamine, 100 U/mL penicillin and 100 µg/mL streptomycin

Abbr.: ATCC, American Type Culture Collection; NIAID, NIH, NIH HIV Reagent Program, Division of AIDS; PBMC, peripheral blood mononuclear cells; RPMI, Roswell Park Memorial Institute 1640 Medium; DMEM, Dulbecco's Modified Eagle Medium; PMID, PubMed Identification

## Supplementary References


1. Seibel, N.M., Eljouni, J., Nalaskowski, M.M. & Hampe, W. Nuclear localization of enhanced green fluorescent protein homomultimers. *Anal Biochem* **368**, 95-99 (2007).
2. Wilen, C.B., Tilton, J.C. & Doms, R.W. HIV: cell binding and entry. *Cold Spring Harb Perspect Med* **2** (2012).
3. Mondor, I., Ugolini, S. & Sattentau, Q.J. Human immunodeficiency virus type 1 attachment to HeLa CD4 cells is CD4 independent and gp120 dependent and requires cell surface heparans. *J Virol* **72**, 3623-3634 (1998).
4. St-Pierre, C. *et al.* Host-soluble galectin-1 promotes HIV-1 replication through a direct interaction with glycans of viral gp120 and host CD4. *J Virol* **85**, 11742-11751 (2011).
5. Clapham, P.R., McKnight, Á., Talbot, S. & Wilkinson, D. HIV entry into cells by CD4-independent mechanisms. *Perspectives in Drug Discovery and Design* **5**, 83-92 (1996).
6. Tsukamoto, T. & Okada, S. The use of RetroNectin in studies requiring in vitro HIV-1 infection of human hematopoietic stem/progenitor cells. *J Virol Methods* **248**, 234-237 (2017).

7. O'Doherty, U., Swiggard, W.J. & Malim, M.H. Human immunodeficiency virus type 1 spinoculation enhances infection through virus binding. *J Virol* **74**, 10074-10080 (2000).
8. Guo, J., Wang, W., Yu, D. & Wu, Y. Spinoculation triggers dynamic actin and cofilin activity that facilitates HIV-1 infection of transformed and resting CD4 T cells. *J Virol* **85**, 9824-9833 (2011).
9. Carter, C.C. *et al.* HIV-1 infects multipotent progenitor cells causing cell death and establishing latent cellular reservoirs. *Nat Med* **16**, 446-451 (2010).
