# Supplementary material for: HIV-1-induced nuclear invaginations mediated by VAP-A, ORP3, and Rab7 complex explain infection of activated T cells
Source: Nat Commun. 2023 Aug 10;14:4588. doi: 10.1038/s41467-023-40227-8 (PMC10415338; doi:10.1038/s41467-023-40227-8)
Supplement: Supplementary file 3 — Description of Additional Supplementary Files [file 41467_2023_40227_MOESM3_ESM.pdf]

Description of Additional Supplementary Files

Supplementary Movie 1.: Colocalization of HIV-1 IN and Rab5-RFP in early endosomes.
Description: HeLa cells expressing Rab5-RFP (red) were infected with HIV-Gag-iGFP for 15 minutes prior to immunolabeling for HIV-1 integrase (IN) using IN-2 antibody (green). Serial x-y sections (0.3 µm each) from bottom to top of cells were acquired by confocal laser scanning microscopy (CLSM), and a 3D image was rendered using Imaris software. A wide area depicting colocalization of IN-2 with Rab5-RFP is shown. (Format mp4; size 5.1 MB).

Supplementary Movie 2.: Colocalization of HIV-1 IN and Rab7-RFP in late endosomes.
Description: HeLa cells expressing Rab7-RFP (red) were infected with HIV-Gag-iGFP for 45 minutes prior to immunolabeling for HIV-1 IN using IN-2 antibody (green). Serial x-y sections (0.3 µm each) from bottom to top of cells were acquired by CLSM, and a 3D image was rendered using Imaris software. A wide area depicting colocalization of IN-2 with Rab7-RFP is shown. (Format mp4; size 6.0 MB).

Supplementary Movie 3: Colocalization of HIV-1 p24 and Rab7 in late endosomes.
Description: HeLa cells were infected with HIV-Gag-iGFP for 1 hour prior to double immunolabeling for HIV-1 p24 (green) and Rab7 (red). Serial x-y sections (0.4-µm each) from top to bottom of cells were acquired by CLSM, and a 3D image was rendered using Imaris software. Note the colocalization of p24 and Rab7. (Format mp4; size 4.3 MB).

Supplementary Movie 4: $Rab7^+$ late endosomes are present in NEI, but not $Lamp1^+$ lysosomes.
Description: HeLa cells were infected with HIV-Gag-iGFP for 1 hour prior to double immunolabeling for Rab7 (red) and Lamp1 (pseudo colored in green for easier visualization). Nuclei were counterstained with DAPI (blue). Serial x-y sections (0.4-µm each) from top to bottom of cells were acquired by CLSM, and a 3D image was rendered using Imaris software. Midway through the video, a cross-sectional cut was introduced to expose the NEI. Note that only $Rab7^+$ late endosomes are present in NEI, whereas $Lamp1^+$ lysosomes are excluded. A single x-y optical section of this video is shown in Fig. 2e. (Format mp4; size 7.1 MB).

Supplementary Movie 5: $Rab7^+$ late endosomes containing HIV-1 IN penetrated into nuclear envelope invagination.
Description: HeLa cells expressing Rab7-RFP (red) were infected with HIV-Gag-iGFP for 1 hour before double immunolabeling for HIV-1 IN using IN-2 antibody (green) and SUN2 (blue). Serial x-y sections (0.3 µm each) from bottom to top of cells were acquired by CLSM and a 3D image was rendered using Imaris software. Note the penetration of IN-2 and Rab7-FRP into NEI. A still image of this video is shown in Supplementary Fig. 6a. (Format mp4; size 8 MB).

Supplementary Movie 6: Presence of late endosomes containing HIV-1 IN in nuclear envelope invagination.

Description: HeLa cells expressing Rab7-RFP (red) were infected with HIV-Gag-iGFP for 1 hour before double immunolabeling for HIV-1 IN using IN-2 antibody (green) and SUN2 (blue). Serial x-y sections (0.3 µm each) from bottom to top of cells were acquired by CLSM and a 3D image was rendered using Imaris software. A sliced nucleus (0.9 µm) shows IN-2 and Rab7-RFP within a transverse section of the NEI. A still image of this video is shown in Supplementary Fig. 6b. (Format mp4; size 6.1 MB).
