# Supplementary material for: HIV-1-induced nuclear invaginations mediated by VAP-A, ORP3, and Rab7 complex explain infection of activated T cells
Source: Nat Commun. 2023 Aug 10;14:4588. doi: 10.1038/s41467-023-40227-8 (PMC10415338; doi:10.1038/s41467-023-40227-8)
Supplement: Supplementary file 10 — Reporting Summary [file 41467_2023_40227_MOESM10_ESM.pdf]

nature portfolio

Corresponding author(s): Aurelio Lorico (alorico@touro.edu) and Denis Corbeil (denis.corbeil@tu-dresden.de)

Last updated by author(s): Jul 5, 2023

Reporting Summary

Nature Portfolio wishes to improve the reproducibility of the work that we publish. This form provides structure for consistency and transparency in reporting. For further information on Nature Portfolio policies, see our Editorial Policies and the Editorial Policy Checklist.

Statistics

For all statistical analyses, confirm that the following items are present in the figure legend, table legend, main text, or Methods section.

n/a	Confirmed	
☐	☒	The exact sample size (*n*) for each experimental group/condition, given as a discrete number and unit of measurement
☐	☒	A statement on whether measurements were taken from distinct samples or whether the same sample was measured repeatedly
☐	☒	The statistical test(s) used AND whether they are one- or two-sided<br>*Only common tests should be described solely by name; describe more complex techniques in the Methods section.*
☒	☐	A description of all covariates tested
☒	☐	A description of any assumptions or corrections, such as tests of normality and adjustment for multiple comparisons
☐	☒	A full description of the statistical parameters including central tendency (e.g. means) or other basic estimates (e.g. regression coefficient) AND variation (e.g. standard deviation) or associated estimates of uncertainty (e.g. confidence intervals)
☐	☒	For null hypothesis testing, the test statistic (e.g. *F*, *t*, *r*) with confidence intervals, effect sizes, degrees of freedom and *P* value noted<br>*Give P values as exact values whenever suitable.*
☒	☐	For Bayesian analysis, information on the choice of priors and Markov chain Monte Carlo settings
☒	☐	For hierarchical and complex designs, identification of the appropriate level for tests and full reporting of outcomes
☐	☒	Estimates of effect sizes (e.g. Cohen's *d*, Pearson's *r*), indicating how they were calculated

Our web collection on statistics for biologists contains articles on many of the points above.

Software and code

Policy information about availability of computer code

Data collection	The average size and concentration of extracellular vesicles used in the study were determined by nanoparticle tracking analysis using ZetaView (version 8.05.12 SP1; Particle Metrix GmbH). All microscopy images were acquired under the same settings using the Nim-OS software (version 1.18) of the Nanoimager microscope (Oxford Nanoimaging, ONI). Flow cytometry data were collected using the CytExpert software (version 2.3; Beckman Coulter). All Western blot data were collected using the iBright FL1000 imaging system (version 1.7.0; Thermo Fisher Scientific).
Data analysis	The average size and concentration of extracellular vesicles used in the study were determined by nanoparticle tracking analysis using ZetaView (version 8.05.12 SP1; Particle Metrix GmbH). All microscopy images were analyzed using Fiji ImageJ (version 1.52p; http://fiji.sc/wiki/index.php/Fiji). For 3D image rendering and analysis, the Imaris software (version 9.6; Bitplane) was utilized. Flow cytometry data were analyzed using FlowJo software (version 10.7.2; BD Life Sciences). Western blot data were analyzed using the cloud-based iBright Analysis software (version 5.2.0; Thermo Fisher Scientific). All statistical analysis were done using Prism 8 (version 8.4.3; GraphPad).

For manuscripts utilizing custom algorithms or software that are central to the research but not yet described in published literature, software must be made available to editors and reviewers. We strongly encourage code deposition in a community repository (e.g. GitHub). See the Nature Portfolio guidelines for submitting code & software for further information.

Data

Policy information about availability of data

All manuscripts must include a data availability statement. This statement should provide the following information, where applicable:

- Accession codes, unique identifiers, or web links for publicly available datasets
- A description of any restrictions on data availability
- For clinical datasets or third party data, please ensure that the statement adheres to our policy

Some proteins were genetically modified to either express a fused fluorescent protein or silence their expression. The human VAP-A (GenBank Accession Number NM_194434.2) and CD9 (GenBank Accession Number NM_001769) were fused to the green fluorescent protein, GFP. To silence specific genes, short hairpin RNA (shRNA) plasmids targeting VAP-A (Accession No. NM_003574.5), VAP-B (Accession No. NM_004738.3), and ORP3 (Accession No. NM_015550.2) were utilized. A comprehensive review of extracellular vesicle characteristics used in the study are available in the EV-TRACK knowledgebase (EV-TRACK, https://evtrack.org/; ID: EV210180, author: Santos).

Human research participants

Policy information about studies involving human research participants and Sex and Gender in Research.

Reporting on sex and gender	*Use the terms sex (biological attribute) and gender (shaped by social and cultural circumstances) carefully in order to avoid confusing both terms. Indicate if findings apply to only one sex or gender; describe whether sex and gender were considered in study design whether sex and/or gender was determined based on self-reporting or assigned and methods used. Provide in the source data disaggregated sex and gender data where this information has been collected, and consent has been obtained for sharing of individual-level data; provide overall numbers in this Reporting Summary. Please state if this information has not been collected. Report sex- and gender-based analyses where performed, justify reasons for lack of sex- and gender-based analysis.*
Population characteristics	*Describe the covariate-relevant population characteristics of the human research participants (e.g. age, genotypic information, past and current diagnosis and treatment categories). If you filled out the behavioural & social sciences study design questions and have nothing to add here, write "See above."*
Recruitment	*Describe how participants were recruited. Outline any potential self-selection bias or other biases that may be present and how these are likely to impact results.*
Ethics oversight	*Identify the organization(s) that approved the study protocol.*

Note that full information on the approval of the study protocol must also be provided in the manuscript.

Field-specific reporting

Please select the one below that is the best fit for your research. If you are not sure, read the appropriate sections before making your selection.

☒ Life sciences ☐ Behavioural & social sciences ☐ Ecological, evolutionary & environmental sciences

For a reference copy of the document with all sections, see nature.com/documents/nr-reporting-summary-flat.pdf

Life sciences study design

All studies must disclose on these points even when the disclosure is negative.

Sample size	All experiments were done in vitro wherein at least 50 cells per experiment were analyzed. No statistical method was used to predetermine sample size. The authors determined that a large sample size of at 50 cells per replicate (each experiment were repeated at least three times) is sufficient to produce significant statistical comparisons.
Data exclusions	No data exclusion was utilized.
Replication	All experiments were performed at least three times, with each being independent biological replicates. All replication attempts were included in the analysis, and were considered successful and reproducible.
Randomization	Since all experiments were done in vitro and utilized cultured cells, randomization does not apply to this study. Instead, experiments were repeated multiple times with each replicate counting toward the analysis.
Blinding	Since no human subjects were involved and all experiments were done in vitro, blinding does not apply to this study. All authors were involved in data collection and analysis.

Reporting for specific materials, systems and methods

We require information from authors about some types of materials, experimental systems and methods used in many studies. Here, indicate whether each material, system or method listed is relevant to your study. If you are not sure if a list item applies to your research, read the appropriate section before selecting a response.

Materials & experimental systems

n/a	Involved in the study
☐	☒ Antibodies
☐	☒ Eukaryotic cell lines
☒	☐ Palaeontology and archaeology
☒	☐ Animals and other organisms
☒	☐ Clinical data
☒	☐ Dual use research of concern

Methods

n/a	Involved in the study
☒	☐ ChIP-seq
☐	☒ Flow cytometry
☒	☐ MRI-based neuroimaging

Antibodies

Antibodies used

All antibodies used in this study were reported in the Methods and Supplementary Table 1. The latter contains their dilution used for different techniques and their validation (see below). Thus, the following antibodies were used throughout the manuscript. Catalog number (cat#), lot number (lot#), clone (if applicable) and supplier are provided. Anti-HIV-1 IN (cat# sc-69721, lot# B0215, clone IN-2), anti-ORP3 (cat# sc-398326, lot# J0419, clone D-12), anti-actin (cat# sc-8432, lot# C1919, clone C-2), and anti-SUN2 (cat# sc-515330, lot# C2416, clone A-10) mouse monoclonal antibodies were purchased from Santa Cruz Biotechnology. Anti-SUN2 (cat# PA5-51539, lot# WD3250552, clone ARC2311) rabbit monoclonal antibody was from Thermo Fisher Scientific. Anti-ORP3 (cat# A304-557A, lot# 1), VAP-A (cat# A304-366A, lot# 1) and VAP-B (cat# A302-894A, lot# 1) rabbit polyclonal antibodies were obtained from Bethyl Laboratories. Anti-lamin B1 (cat# ab8982, lot#GR216605-2, clone 119D5-F1), anti-HIV-1 p24 (cat# ab63958, lot# GR3253964-11, clone 5) and anti-LAMP1 (cat# ab25630, lot# GR3444510-1, clone H4A3) mouse monoclonal, as well as anti-Rab7 (cat# ab137029, lot# GR155792-43, clone EPR7589), anti-CD63 (cat# ab252919, lot# GR3270794-4, clone EPR22458-280), and anti-LAMP1 (cat# ab278043, lot# GR3430890-5, clone EPR24395-31) rabbit monoclonal antibodies were purchased from Abcam. Anti-GAPDH (cat# NB300-326, lot# A1) rabbit polyclonal antibody was purchased from Novus Biologicals. Phycoerythrin (PE)-conjugated anti-CD3 (cat# 12-0039-42, lot#, 2350761, clone HIT3a) and Brilliant Violet 421 (BV421)-conjugated anti-CD4 (cat# 317434, lot# B344244, clone OKT4) mouse monoclonal antibodies were purchased Thermo Fisher Scientific and BioLegend, respectively. RD1-conjugated anti-Gag (cat# 6604667, lot# 7433116, clone KC57) mouse monoclonal antibodies were purchased from Beckman Coulter. Fluorescein (FITC)-conjugated donkey anti-mouse (cat# 715-095-150, lot# 129233) and anti-rabbit (cat# 711-095-152, lot# 140503) IgG and tetramethylrhodamine donkey anti-rabbit IgG (cat# 711-025-152, lot# 143041) were obtained from Jackson ImmunoResearch Laboratories. Alexa Fluor647-conjugated goat anti-mouse (cat# A-21237, lot# 2105224) and anti-rabbit (cat# A-21246, lot# 2098531) IgG and Alexa Fluor488-conjugated goat anti-mouse (cat# A-11017, lot# 2108802) and anti-rabbit (cat# A-11070, lot# 2160404) IgG were purchased from Thermo Fisher Scientific. DyLight® 550-conjugated goat anti-mouse (cat# ab98758, lot# GR3252344-3) IgG was obtained from Abcam.

Validation

All primary antibodies are validated in their application use by the manufacturer. The species and manufacturer are provided above. Application such as immunocytochemistry (ICC), immunoisolation (IS), immunoblotting (IB), and/or flow cytometry (FC) are indicated in parenthesis, while validation, either provided as PubMed Identifier (PMID) and/or figure references from the manuscript, is listed subsequently. Starting with all mouse antibodies used in the study, anti-HIV-1 IN Ab (ICC and IB) is validated in PMID 30804369 and 25808736 and in Figures 1a, b, g, j, k, l; 2a, b, c, g; 3a; 4c, d, g; 6i, k; 8d, e; and Supplementary Figures 2b; 4c; 6; 10f; 11a, b, d, e; and 15a, c. Anti-ORP3 Ab (ICC, IS, and IB) is validated in PMID 34429859 and in Figures 7a,b; 8h; 9e; and in Supplementary Figures 5b and 7a. Anti-actin Ab (IB) is validated in PMID 36003783 and in Supplementary Figure 5. Anti-SUN2 Ab (ICC) is validated in PMID 30982221 and in Figures 1e; 3e; 6a, c, g, l; and Supplementary Figure 14a. Anti-Lamin B1 Ab (ICC and IB) is validated in PMID 33767161 and in Figures 4g and 6f. Anti-HIV-1 p24 Ab (ICC) is validated PMID 36062074 and in Supplementary Figures 4 and 10f. Anti-LAMP1 Ab (ICC) is validated in PMID 33731717 and in Figures 2d, e; and 6l. Phycoerythrin-conjugated anti-CD3 Ab (FC) is validated in PMID 32508837 and in Supplementary Figure 13. Brilliant Violet 421-conjugated anti-CD4 Ab (FC) is validated in PMID 32330454 and in Supplementary Figures 10a and 13. RD1-conjugated anti-Gag Ab (FC) is validated in PMID 20208541 and in Figure 5e; and Supplementary Figure 12b, c. Rabbit antibodies are listed next. Anti-SUN2 Ab (ICC) is validated in PMID 32908309 and in Figures 3a; 4d; 6f, l; and Supplementary Figure 6. Anti-ORP3 Ab (IB) is validated in PMID 34429859 and in Figures 3a, e; 4b; 7a, b; 8h, j; 9e; and Supplementary Figure 7a. Anti-VAP-A Ab (ICC and IB) is validated in PMID 34429859 and in Figures 1a, b, e, g; 3a, e; 4b, c, i; 6a, c, g, i; 7a, b; 8a, h; 9e; and Supplementary Figures 2a; 5; 9a; 10f; 11a, b, d, g; and 14a. Anti-VAP-B Ab (ICC and IB) is validated in PMID 34429859 and in Figures 3a, e; and Supplementary Figure 5a. Anti-Rab7 Ab (ICC and IB) is validated in PMID 31085713 and in Figures 2a, d, e, g; 4b; 6k, l; 7a, b; 8d, e, h; 9e; and Supplementary Figures 4a; 7a; and 15. Anti-CD63 Ab (ICC) is validated in PMID 34332599 and in Figure 2b. Anti-LAMP1 Ab (ICC) is validated in PMID 35984564 and in Figure 2c. Anti-GAPDH Ab (IB) is validated in PMID 27602114 and in Figure 4g.

Eukaryotic cell lines

Policy information about cell lines and Sex and Gender in Research

Cell line source(s)

The human cervix epithelioid cell line HeLa (ATCC®CCL-2) and embryonic kidney cell line 293T (CRL-3216) were obtained from the American Type Culture Collection (ATCC), while CD4-positive HeLa cells (ARP-1109, clone 1022) were acquired from NIH HIV Reagent Program, Division of AIDS, NIAID, NIH. Human helper T (CD4+) cells were obtained from Discovery Life Sciences

	(#C4T0015-Z1110032763081618BA) or isolated from healthy volunteers. FEMX-I cell line was originally derived from a lymph node metastasis of a patient with malignant melanoma and cultured in vitro. They were a gift from Dr. Oystein Fodstad.
Authentication	Cell lines obtained from vendors are authenticated per their guidelines. For HeLa and 293T cells from ATCC, they were authenticated by morphology, STR profiling, and karyotyping. For CD4-positive HeLa and CD4-positive helper T cells, they were authenticated by morphology and flow cytometry analysis to determine CD4 expression. FEMX-I cells were authenticated by morphology and proteomics, and are found to be wild type for BRAF, PTEN, and NRAS by gene expression analysis.
Mycoplasma contamination	Cells are negative for mycoplasma contamination. They were regularly verified for absence of mycoplasma contamination by either staining with DAPI and visualization under a fluorescent microscope or polymerase chain reaction using the MycoSEQ™ Mycoplasma Detection Kit (#4460626, Thermo Fisher Scientific).
Commonly misidentified lines (See ICLAC register)	There are no commonly misidentified cell lines used in this study.

Flow Cytometry

Plots

Confirm that:

☒ The axis labels state the marker and fluorochrome used (e.g. CD4-FITC).

☒ The axis scales are clearly visible. Include numbers along axes only for bottom left plot of group (a 'group' is an analysis of identical markers).

☒ All plots are contour plots with outliers or pseudocolor plots.

☒ A numerical value for number of cells or percentage (with statistics) is provided.

Methodology

Sample preparation	HeLa (cell line), CD4-positive HeLa (cell line), or CD4-positive T cells (human donor obtained from Discovery Life Sciences or isolated from healthy volunteers) seeded on 24-well plates were processed for flow cytometry after drug treatments and/or virus infection. Cells were trypsinized (for HeLa cells only) using Trypsin/EDTA solution for 3 minutes, blocked by the addition of respective cell medium, centrifuged at 200 x g for 5 minutes, and resuspended in PBS. They were immediately analyzed on the CytoFlex flow cytometer system (Beckman Coulter). Alternatively, CD4-positive T cells were first immunolabeled with fluorochrome-conjugated anti-CD3 and CD4 antibodies diluted in PBS containing 5% FBS for 45 minutes on ice, washed twice by centrifugation at 300 x g for 10 minutes, and resuspended in PBS containing 5% FBS prior to analysis. In other experiments, cells were fixed, permeabilized, then immunolabeled with fluorochrome-conjugated anti-Gag antibody prior to analysis.
Instrument	CytoFLEX Violet-Blue-Red Series from Beckman Coulter; system ID 59162703
Software	CytExpert, version 2.3, by Beckman Coulter was used to collect data. FlowJo, version 10.7.2, by BD Life Sciences was used to analyze data.
Cell population abundance	No sorting was performed. All samples are of one type of cell population, e.g. HeLa, CD4-positive HeLa or CD4-positive T cells.
Gating strategy	Detector gain for the all channels was kept constant for all experiments. A gate was applied on cell population, as opposed to debris, based on their forward and side scatter characteristics, wherein the gate was drawn around a dense cluster plot. Cells without viral infection or antibody staining were used to gate the negative cell population on the appropriate fluorescent detector channel. CD4-positive T cells were further gated based on both CD3 and CD4 positivity. In some experiments, single (EGFP or Gag) and double positives (EGFP and Gag) are shown in a quadrant plot. At least 100,000 events were acquired.

☒ Tick this box to confirm that a figure exemplifying the gating strategy is provided in the Supplementary Information.
