# Supplementary material for: HIV-1-induced nuclear invaginations mediated by VAP-A, ORP3, and Rab7 complex explain infection of activated T cells
Source: Nat Commun. 2023 Aug 10;14:4588. doi: 10.1038/s41467-023-40227-8 (PMC10415338; doi:10.1038/s41467-023-40227-8)

Figure 4b

IS
1° Ab: ORP3 (A304-557A)
2° Ab: anti-rabbit FITC

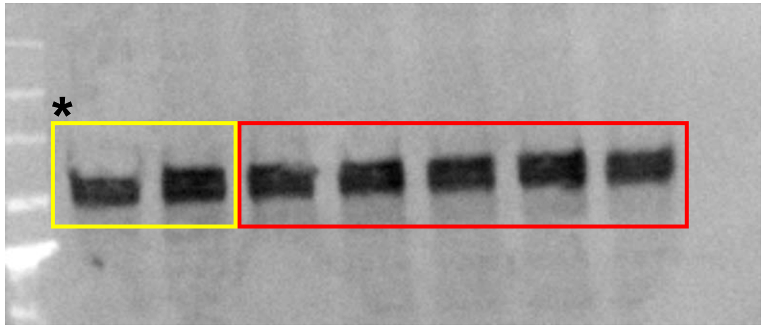

IS
1° Ab: VAP-A (A304-366A)
2° Ab: anti-rabbit FITC

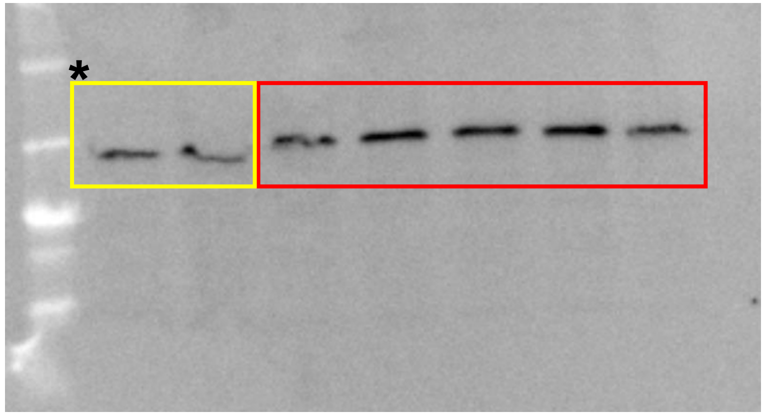

Input
1° Ab: Rab7 (ab137029)
2° Ab: anti-rabbit FITC

IS
1° Ab: Rab7 (ab137029)
2° Ab: anti-rabbit FITC

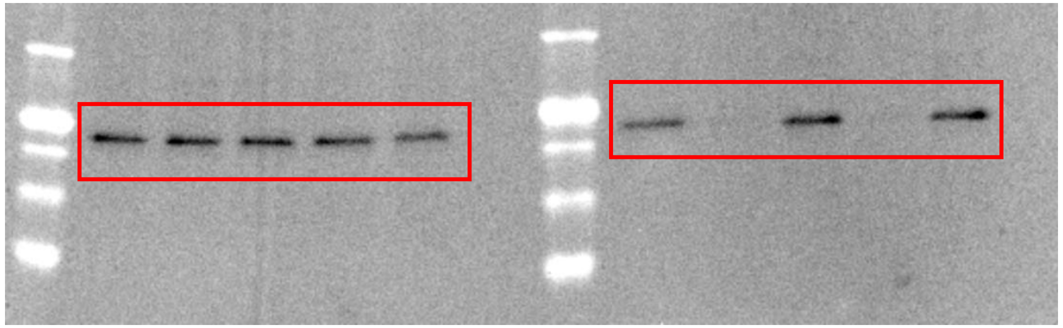

*not used. Input for DMSO and ICZ, respectively

Figure 4g

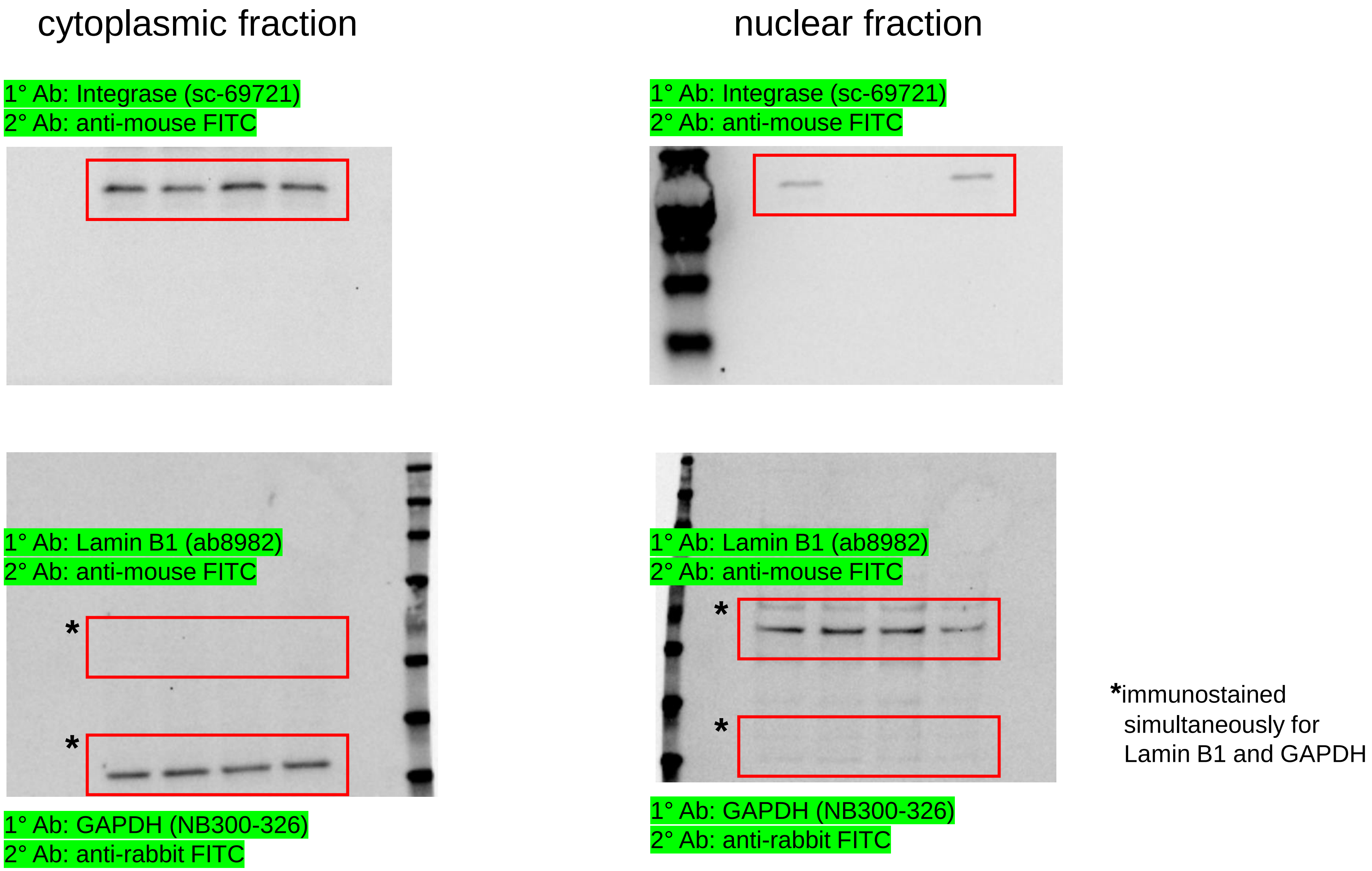
cytoplasmic fraction
nuclear fraction
1° Ab: Integrase (sc-69721)
2° Ab: anti-mouse FITC
1° Ab: Integrase (sc-69721)
2° Ab: anti-mouse FITC
1° Ab: Lamin B1 (ab8982)
2° Ab: anti-mouse FITC
1° Ab: Lamin B1 (ab8982)
2° Ab: anti-mouse FITC
*
*
*
*
1° Ab: GAPDH (NB300-326)
2° Ab: anti-rabbit FITC
1° Ab: GAPDH (NB300-326)
2° Ab: anti-rabbit FITC
*immunostained simultaneously for Lamin B1 and GAPDH

Figure 7a

Input
1° Ab: ORP3 (A304-557A)
2° Ab: anti-rabbit FITC

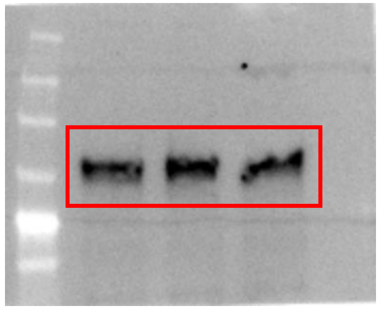

Input
1° Ab: VAP-A (A304-366A)
2° Ab: anti-rabbit FITC

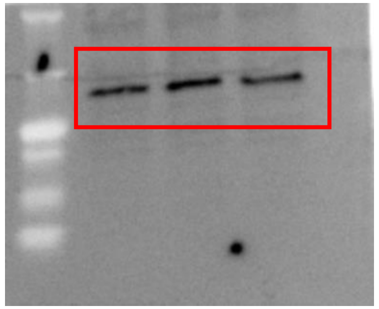

Input
1° Ab: Rab7 (ab137029)
2° Ab: anti-rabbit FITC

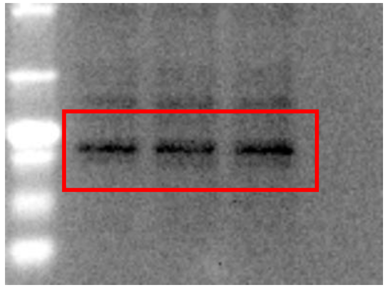

IS
1° Ab: ORP3 (A304-557A)
2° Ab: anti-rabbit FITC

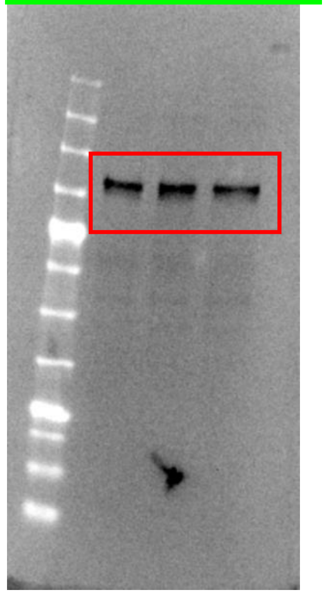

IS
1° Ab: VAP-A (A304-366A)
2° Ab: anti-rabbit FITC

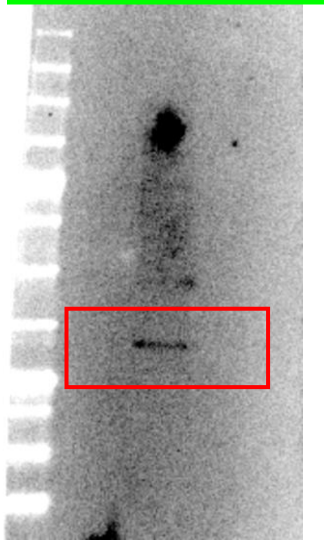

IS
1° Ab: Rab7 (ab137029)
2° Ab: anti-rabbit FITC

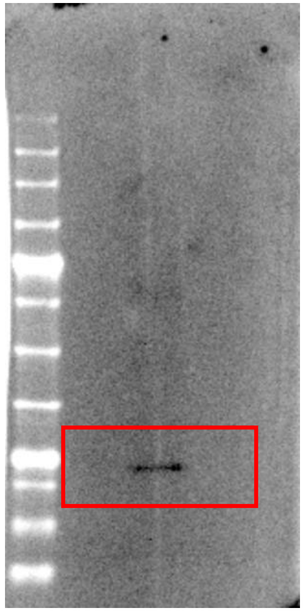

Figure 7b

Input
1° Ab: ORP3 (A304-557A)
2° Ab: anti-rabbit FITC

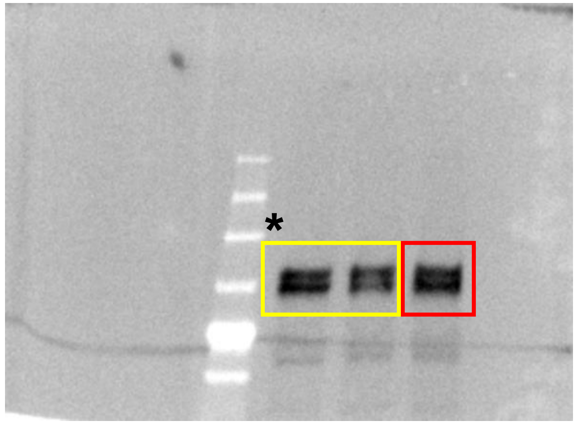

Input
1° Ab: VAP-A (A304-366A)
2° Ab: anti-rabbit FITC

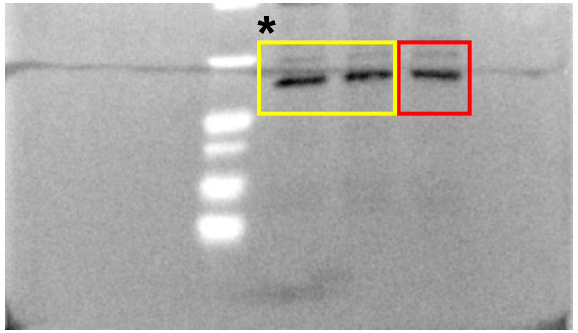

*not shown

Input
1° Ab: Rab7 (ab137029)
2° Ab: anti-rabbit FITC

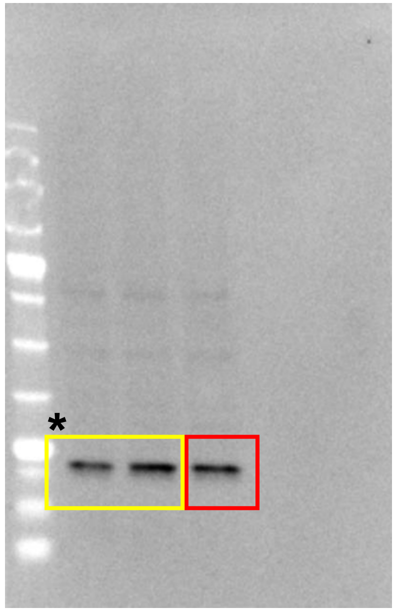

IS
1° Ab: ORP3 (A304-557A)
2° Ab: anti-rabbit FITC

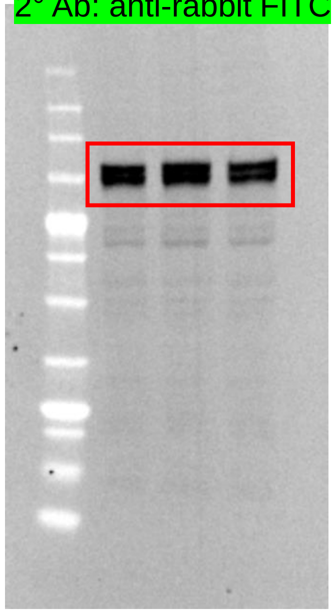

IS
1° Ab: VAP-A (A304-366A)
2° Ab: anti-rabbit FITC

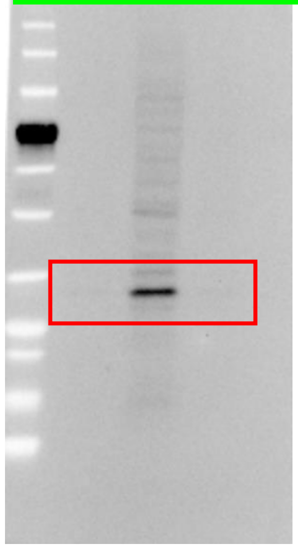

IS
1° Ab: Rab7 (ab137029)
2° Ab: anti-rabbit FITC

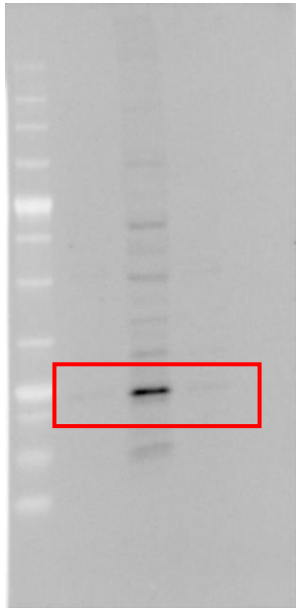

Figure 8h

IS
1° Ab: ORP3 (A304-557A)
2° Ab: anti-rabbit FITC

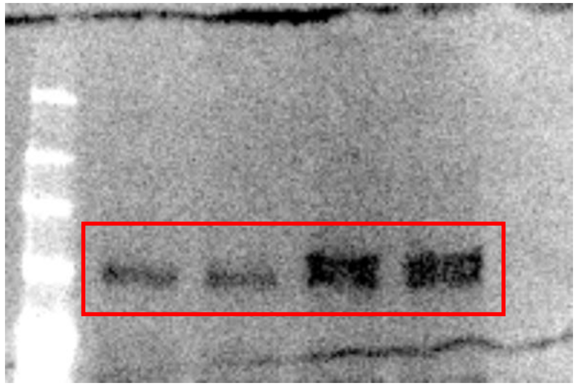

IS
1° Ab: VAP-A (A304-366A)
2° Ab: anti-rabbit FITC

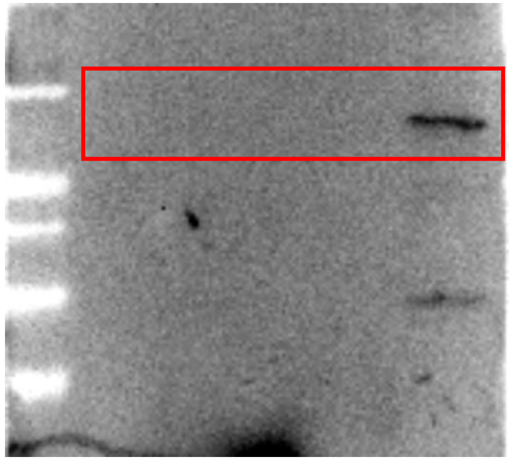

IS
1° Ab: Rab7 (ab137029)
2° Ab: anti-rabbit FITC

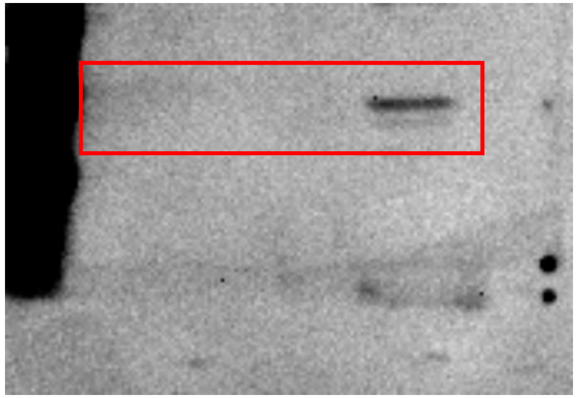

Input
1° Ab: VAP-A (A304-366A)
2° Ab: anti-rabbit FITC

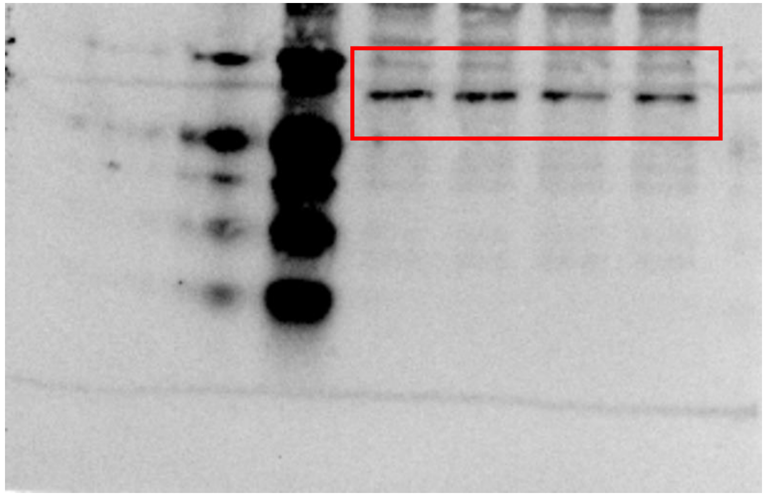

Input
1° Ab: Rab7 (ab137029)
2° Ab: anti-rabbit FITC

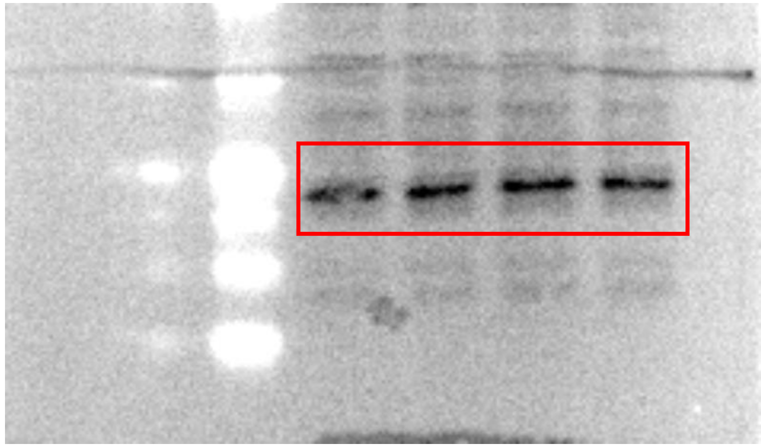

Figure 8j

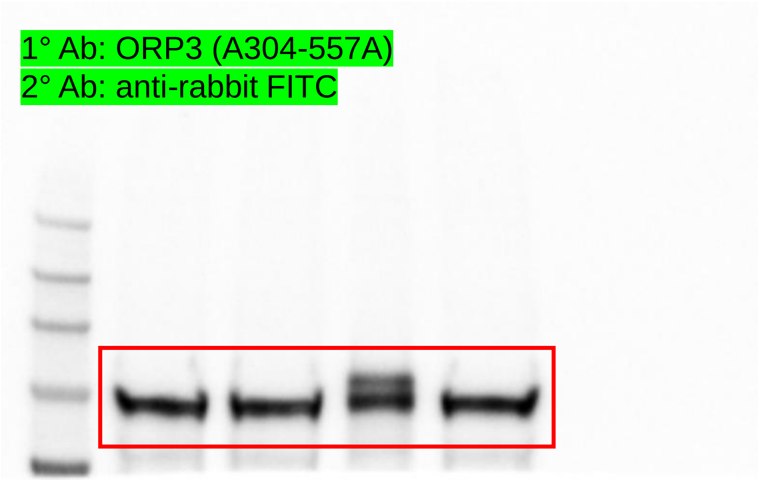

Figure 9e

IS
1° Ab: ORP3 (A304-557A)
2° Ab: anti-rabbit FITC

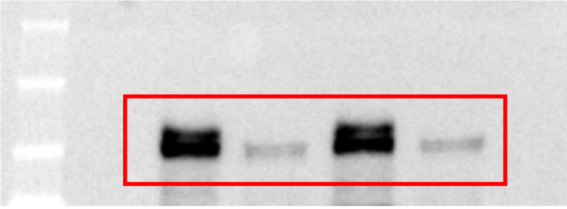

Input
1° Ab: ORP3 (A304-557A)
2° Ab: anti-rabbit FITC

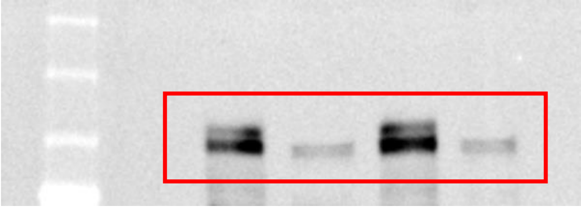

Input
1° Ab: Rab7 (ab137029)
2° Ab: anti-rabbit FITC

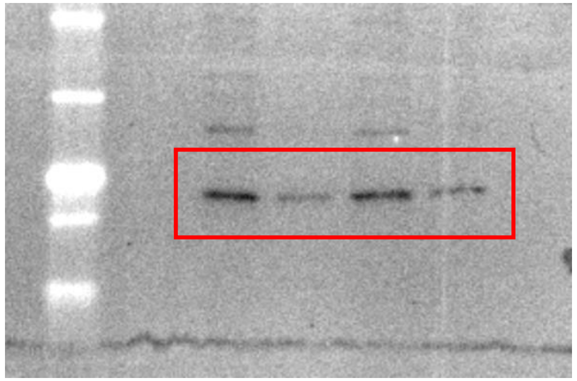

IS
1° Ab: VAP-A (A304-366A)
2° Ab: anti-rabbit FITC

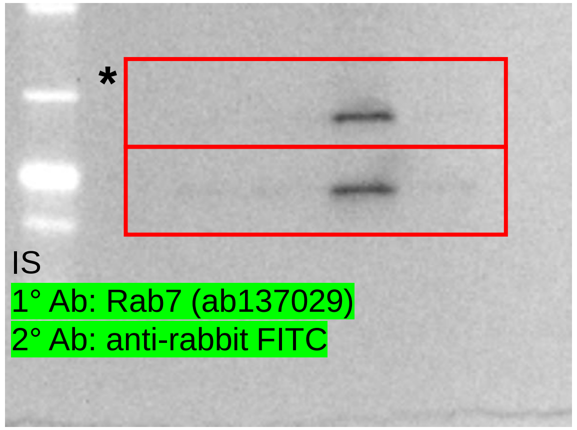

IS
1° Ab: Rab7 (ab137029)
2° Ab: anti-rabbit FITC

Input
1° Ab: VAP-A (A304-366A)
2° Ab: anti-rabbit FITC

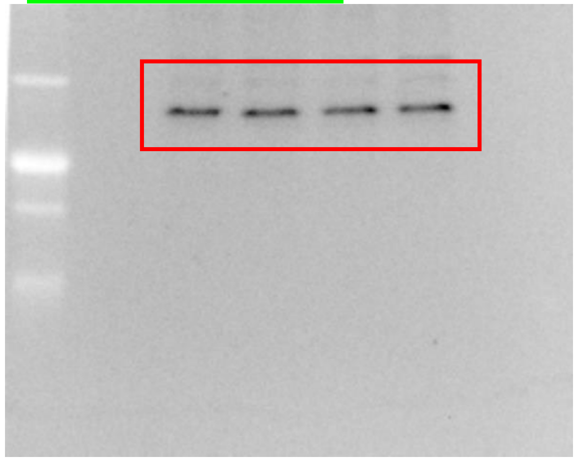

*immunostained simultaneously for
VAP-A and Rab7

Supplementary Figure 5a

left

1° Ab: VAP-A (A304-366A)

2° Ab: anti-rabbit FITC

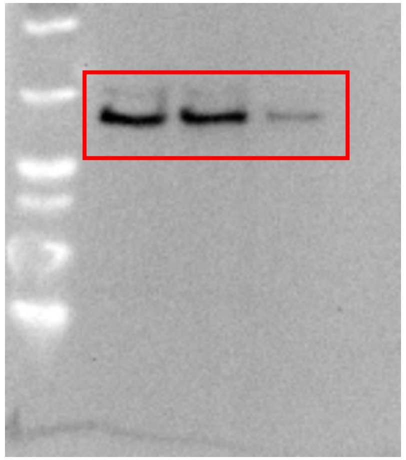

left

1° Ab: VAP-B (A302-894A)

2° Ab: anti-rabbit FITC

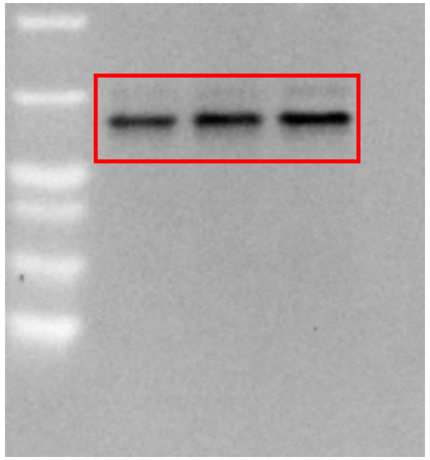

left

1° Ab: Actin (sc-8432)

2° Ab: anti-mouse FITC

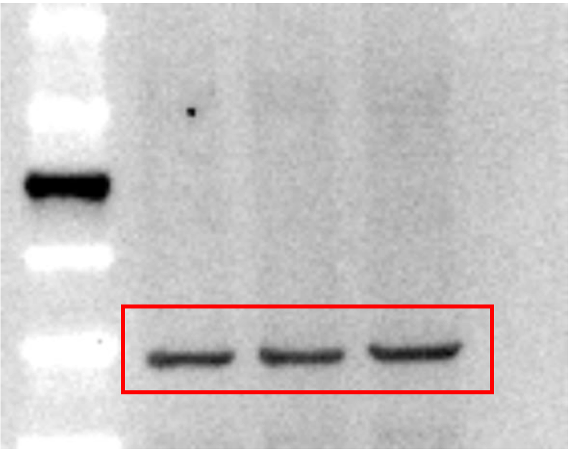

right

1° Ab: VAP-B (A302-894A)

2° Ab: anti-rabbit FITC

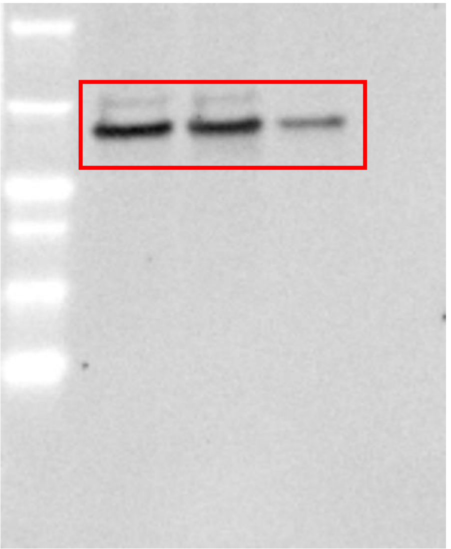

right

1° Ab: VAP-A (A304-366A)

2° Ab: anti-rabbit FITC

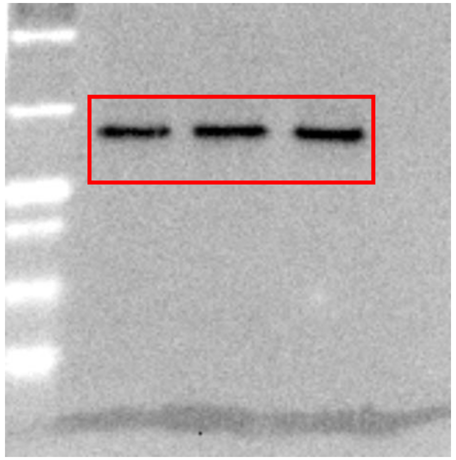

right

1° Ab: Actin (sc-8432)

2° Ab: anti-mouse FITC

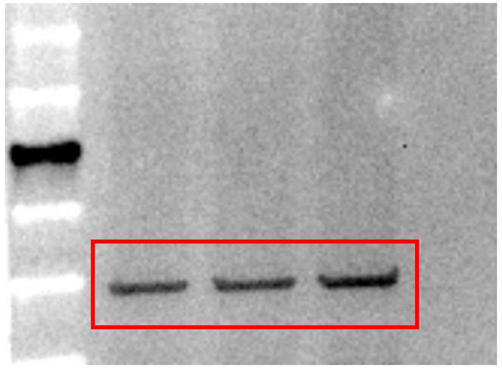

Supplementary Figure 5b

1° Ab: ORP3 (sc-398326)
2° Ab: anti-mouse FITC

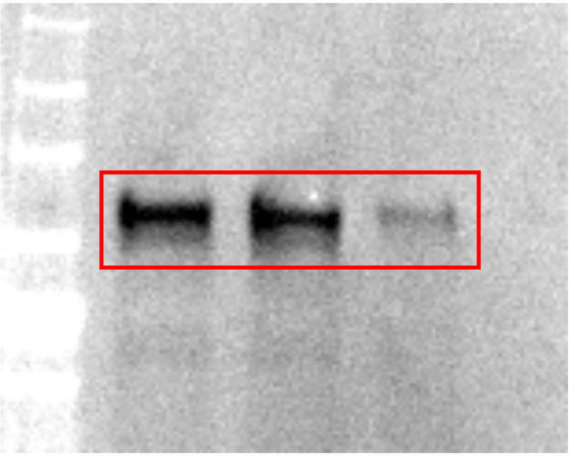

1° Ab: VAP-A (A304-366A)
2° Ab: anti-rabbit FITC

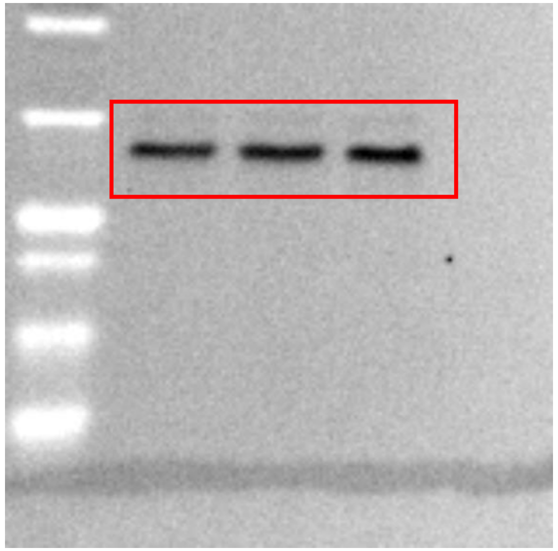

1° Ab: Actin (sc-8432)
2° Ab: anti-mouse FITC

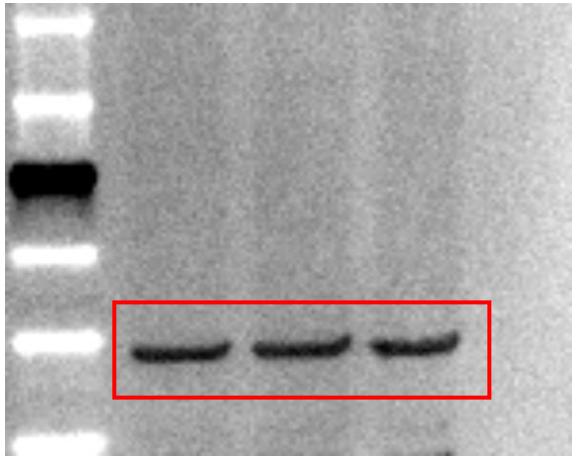

Supplementary Figure 7a

IS
1° Ab: ORP3 (A304-557A)
2° Ab: anti-rabbit FITC

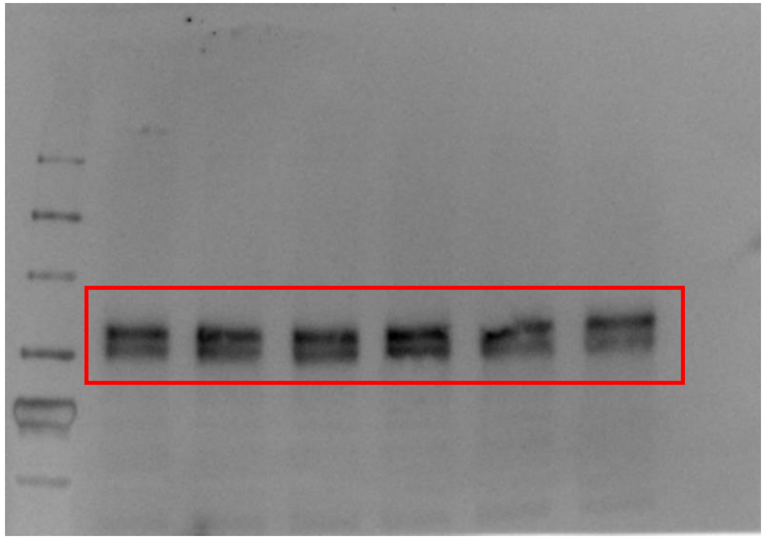

IS
1° Ab: Rab7 (ab137029)
2° Ab: anti-rabbit FITC

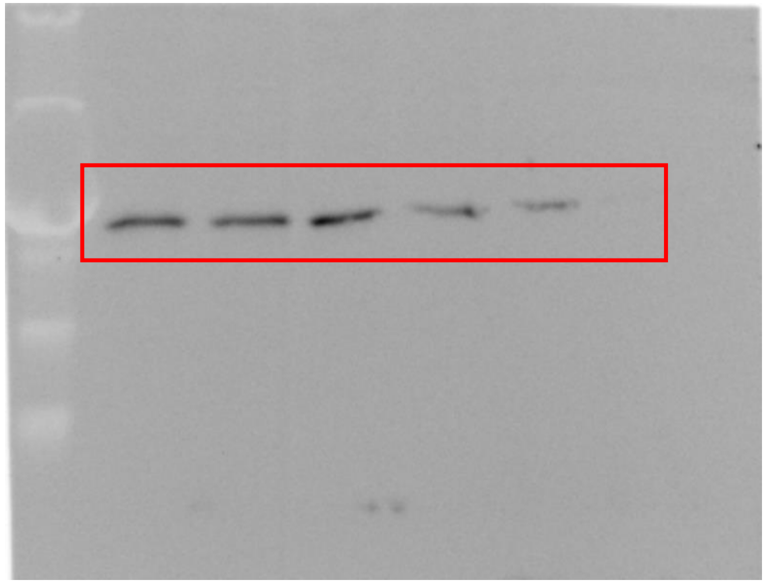

Supplement: Supplementary file 11 — Source Data [file 41467_2023_40227_MOESM11_ESM.zip › Source Data folder/Uncropped blots.pdf]
